# Selecting force plate tests and metrics to complement physical preparation strategies prior to commencing strenuous military training courses

**DOI:** 10.3389/fspor.2026.1891717

**Published:** 2026-08-26

**Authors:** Peter Ladlow, Nicholas J. Ripley, Damjana V. Cabarkapa, Dimitrije Cabarkapa, Dale W. Chapman, Mary Claire Geneau, G. Gregory Haff, Lachlan P. James, Paul Jones, Jason P. Lake, Christopher J. Sole, Anthony Turner, Marina De Vecchis, Natalie Masters, Charles Coussens, Robyn Holton, Russell J. Coppack, Colin Suffield, Paul Comfort

**Affiliations:** 1 Academic Department of Military Rehabilitation (ADMR), Defence Medical Rehabilitation Centre (DMRC) Stanford Hall, Loughborough, United Kingdom; 2 Department for Health, University of Bath, Bath, United Kingdom; 3 Directorate of Psychology and Sport, University of Salford, Salford, United Kingdom; 4 Strength and Power Research Group, Edith Cowan University, Joondalup, WA, Australia; 5 D2 Lab, Novi Sad, Serbia; 6 Faculty of Physical Education and Sports Management, Singidunum University, Belgrade, Serbia; 7 Jayhawk Athletic Performance Laboratory—Wu Tsai Human Performance Alliance, University of Kansas, Lawrence, KS, United States; 8 School of Allied Health, Curtin University, Perth, WA, Australia; 9 School of Exercise Science, Physical & Health Education, University of Victoria, Victoria, BC, Canada; 10 Canadian Sport Institute Pacific, Victoria, BC, Canada; 11 Exercise Medicine Research Institute, Edith Cowan University, Joondalup, WA, Australia; 12 Sport, Performance, and Nutrition Research Group, School of Allied Health, Human Services, & Sport, La Trobe University, Melbourne, VIC, Australia; 13 Chichester Institute of Sport, Nursing, and Allied Health, University of Chichester, Chichester, United Kingdom; 14 Department of Health and Human Performance, The Citadel—The Military College of South Carolina, Charleston, SC, United States; 15 Faculty of Science and Technology, London Sport Institute, Middlesex University, London, United Kingdom; 16 Royal Army Physical Training Corps (RAPTC), Aldershot, United Kingdom; 17 HQ Royal Army Medical Services (RAMS), Staff College, Camberley, United Kingdom

**Keywords:** injury mitigation, load carriage, neuromuscular performance, profiling, tactical athletes

## Abstract

**Background:**

The aim of this study was to seek opinion (via an online survey) and gain consensus of which force plate tests and metrics may be considered by military operating/training staff to better screen physical performance characteristics prior to military personnel attending a physically arduous military training course.

**Methods:**

Ten responders with known experience and/or expertise in using force plate technology for the measurement of force production characteristics completed the survey. Responders were asked to select the most important force plate test and metrics to complement a pre-course physical performance battery to assess physical readiness, musculoskeletal injury risk and likelihood of passing the course. Types of force plate tests included bodyweight tests, single joint and multi-joint isometric tests, countermovement jump (CMJ), squat jump, rebound jump tests and drop landings. For each “type” of test recommended, a drop-down list of metrics was provided for responders to select. These metrics were categorized as “outcome-based”, “force-based”, “power-based”, “rate of force development-based”, “impulse-based”, “velocity-based”, and “time-based” metrics. If answered “yes”, a comprehensive and standardized list of force plate derived metrics would appear. Responders were asked to select metrics from this list that they considered important to report.

**Results:**

Initially, there was a strong consensus on “test” selection (multi-joint isometrics test, CMJ, and drop jump), but not “metric” selection.

**Discussion:**

Responder metrics were evaluated for biomechanical relevance, redundancy, reliability, test duration, interpretability and feasibility before consensus was reached for minimum force plate tests and metric dataset to complement physical readiness strategies prior to commencing strenuous military training courses.

## 1 Introduction

Assessing physical performance characteristics in military personnel is common practice (1–3) and each branch of the military has their own physical employment standards (PES). The primary objective for assessing individuals against these PES is to help determine whether individuals are prepared physically for the demands of their occupational role (4, 5). However, these standards are generally designed to determine whether personnel can complete critical and physically demanding occupational tasks to a minimum acceptable level, often under a defined reasonable worst-case scenario (6). They do not provide a detailed profile of the neuromuscular strategies or physical capacities underpinning that task performance.

Military occupations are commonly categorized as ground close combat (GCC) and non-GCC roles. GCC roles include infantry personnel who often have higher PES compared to other military roles (7). Some specialist infantry units require service personnel to complete more physically arduous selection courses compared to “regular” infantry units (2). Examples of specialist infantry units from the UK military include Army Paratroopers, Royal Marine Commandos, and Special Forces. Exemplifying highly stressful military training events, these training/selection courses span several weeks to months in duration and are designed to assess potential candidates' ability to operate under extreme and unpredictable environments (8, 9). The prevalence of voluntary withdrawal due to an inability to tolerate the physical demands of these courses and/or musculoskeletal injury are common. Whilst ensuring the highest of physical standards are met, maximizing the number of personnel who pass these types of elite unit courses remain a key objective to Defense organizations globally. These courses are delivered with substantial staff burden and at great economic cost. It is therefore imperative that (1) service personnel arrive physically prepared for the demands of the course, and (2) a comprehensive screening procedure is in place prior to the course to ensure training/operating staff are provided the greatest opportunity to identify individual service personnel at increased risk of musculoskeletal injury (MSKI) or course failure, and those with a moderate to high chance of successfully passing. Such pre-course screening procedures typically include psychological, tactical/skill-based and physical assessments. However, the decision-making value of such screening depends on the validity of both the tests selected and the standards applied to their interpretation.

Physical characteristics are typically assessed using field-based assessments which are inexpensive and quick to administer on mass (7). Examples include, predicted one repetition maximum strength assessment (e.g., a hexagonal barbell deadlift), strength endurance task [e.g., maximal number of press-ups in a pre-determined time (acknowledging that timed adds an element of power)], jump assessment (e.g., a broad jump) or a timed best effort run (with or without load carriage) (1, 2, 10). A fundamental limitation of using these tests is that such methods restrict practitioners to a single outcome measure, (e.g., jump distance or height) (11). Although this metric can be useful, it fails to provide valuable information about movement strategies, which may be more sensitive to training loads (12–14). For example, jump height can be preserved even with a change in movement strategy (i.e., an increase in time to take-off, or propulsion phase duration) in order to maintain propulsive impulse, even when propulsive force decreases (14) or due to a decrease in body mass (15), which permits a reduction in net propulsive impulse, as it is the relative net impulse which determines velocity at take-off and therefore jump height (16, 17).

Success in strenuous military selection courses is multifactorial, determined by an interplay of demographic, psychological, and physical variables. However, physical performance (i.e., composite scores collected using push-ups, pull-ups, sit-ups, a two-mile run, loaded march) has previously been reported to be the most predictive variable of successful United States Special Forces selection (8). Measuring force–time characteristics, via force plate technology, may complement existing screening protocols by facilitating a more comprehensive evaluation of neuromuscular performance than recording the physical outcome (e.g., jump height or predicted one repetition maximum alone) (7, 11, 18). Muscle strength is the strongest predictor of non-contact MSKI in athletes (19–21) and therefore may be predictive of injury risk during arduous military training, and as such highly reliable measures of strength such as the isometric midthigh pull (IMTP) (22–24) rather than predictions of strength should be utilized. For example, in Canadian armed forces, uninjured personnel demonstrated higher relative peak force in the IMTP (25) indicating the potential protective effect of higher strength levels. In addition, IMTP peak force has been reported to be associated with casualty drag performance (26, 27). However, other force-time variables are rarely reported in this context and could provide greater insight.

The use of force plates in military settings is growing rapidly and there are examples in the literature of this technology being used on large samples of military personnel (>500 recruits/cadets) (28–33). These investigations have predominantly used countermovement jumps or multi-joint isometric assessments to describe neuromuscular performance, compare subgroups, examine associations with occupational or physical performance and monitor changes across military training (11). Collectively, they demonstrate that force plate testing can be implemented at scale, although test selection, processing procedures and metric reporting have varied substantially across studies.

The assessment of force production characteristics is important in relation to physical performance as it is force relative to the mass being accelerated that determines acceleration, with the duration of force production is limited during many tasks, highlighting the importance of also evaluating rapid force production. Additionally, deceleration is determined by the impulse (i.e., *Δ*force × *Δ*time) generated by the individual based on the impulse momentum relationship. The assessment of maximal and rapid force production characteristics using force plates offers the practitioner insights into neuromuscular performance of personnel which are not attainable through conventional field-based testing. However, the results of a recent scoping review (11) revealed that research groups frequently underutilize this technology, with most researchers [31 studies (77.5%) of the 40 articles included] only using one force plate test to determine the force generating capacity of their military population of interest, which is unlikely to provide a complete profile of the individuals maximal and rapid force generating capacity. Moreover, if only one test is utilized, only isometric, slow (>250 ms) stretch shortening cycle (SSC) or fast (<250 ms) SSC function can be evaluated, rather than the best practice of providing the complete picture of force production characteristics from all three. Such an approach to utilizing different tests to evaluate different strength qualities has recently been recommended, to ensure comprehensive evaluation of force production characteristics (34–36). In addition, the results of the scoping review highlighted that no more than two metrics from each test were commonly reported (11). For example, two or fewer metrics were used in 70% of IMTP tests and 60% of countermovement jump (CMJ) tests, which for the CMJ would not provide a complete profile of the individuals output (i.e., jump height) and movement strategy [e.g., countermovement depth, or propulsive phase duration and relative net propulsive force (i.e., relative net propulsive impulse which determines velocity at take-off and therefore jump height)] used to achieve this output. Considering there are multiple force plate test options and hundreds of metrics available, this highlights substantial knowledge gaps in how force plate technology may be best integrated into Defense organizations. Bridging this gap would inform military policy and practices by facilitating the design and implementation of optimal physical preparation and injury mitigation strategies.

Selecting the correct force plate tests and metrics is crucial across research and in applied settings. The large quantity of metrics provided by commercial force plate providers can make identifying the most important metrics to use during neuromuscular performance test batteries quite daunting to the end-user (37). However, some can easily be removed from consideration as they are duplicates (e.g., time to take-off, movement time, contraction time), or incorrectly termed (e.g., rate of velocity development, which is acceleration). Future applied and research practices could be informed by evaluating the opinion of experienced force plate users. Generic recommendations on CMJ metric selection across athletic populations have been published (38), but no force plate test or metric selection recommendations have been shared that might influence military performance policy and practices, such as the prediction of MSKI occurrence or pass criteria. Unlike many athletic populations, military personnel are required to sustain physical performance under conditions of sleep deprivation, energy deficit, prolonged load carriage, environmental stress, and cumulative fatigue. Furthermore, military performance outcomes extend beyond maximal athletic output and include resilience to musculoskeletal injury, sustained operational performance, and tolerance to repeated occupational loading. Consequently, force plate testing should not be considered a replacement for established assessments of aerobic fitness, maximal strength, muscular endurance, load carriage or occupational task performance. The potential contribution of force plate testing is to provide task-specific information concerning the magnitude, timing and strategy of force production that cannot be inferred from a single outcome such as jump height or one repetition maximum. Whether this additional information improves prediction or physical-preparation decisions beyond conventional tests remains to be established in military populations. No previous study has attempted to establish expert opinion regarding which force plate tests and metrics should be prioritized specifically within a military screening context. Existing military force plate literature demonstrates substantial heterogeneity in both test selection and metric reporting. If recommendations from experienced force plate users were to be implemented, this could provide guidance to training/operating staff surrounding entry criteria/standards and suitability of service personnel wishing to attend future elite unit courses (11). Therefore, the aim of this study was to identify an expert-informed minimum set of force plate tests and metrics that could be used during the pre-course preparation period to characterise neuromuscular performance and complement existing physical-readiness strategies before service personnel commence physically arduous military training courses.

## 2 Methods

### 2.1 Experimental approach to the problem

Google Forms were used to administer the online survey. Invitations were sent via email, alongside a participant information sheet and explanatory statement. All responders provided electronic informed consent. Recruitment took place between 29 June and 18 August 2025. The cross-sectional survey started by asking short questions about the responders' personal background, including employment history and experience with force plate technology, before seeking opinion of specific force plate test and metric selection. This was a single-entry anonymized survey. Responders were not permitted to share the survey link with others. This study was ethically approved by the University of Salford Research Ethics Committee (Reference: 7289). The article is reported following the consensus-based checklist for reporting of survey study (CROSS) guidelines (39).

The study was not a formal Delphi study. Instead, it used a modified expert-survey and synthesis process. The survey was used to identify tests and metrics considered important by experienced force plate users, after which the findings were synthesised with established biomechanical principles, consideration of metric selection and redundancy, available military evidence and practical constraints associated with testing large military cohorts.

The survey was developed from the findings of the preceding military force plate scoping review, supplemented by current force plate literature and known metric availability. The survey was pre-tested on two occasions by PL, NJR and PC, who had experience in force plate testing comparable with the intended responder population. Pre-testing examined item clarity, completeness of test and metric options, consistency of terminology, branching logic, duplication and technical functionality. No items were revised after pilot testing. The survey consisted of 12 sections and 144 questions. Independent external content validation was not completed before survey administration because the identified external specialists comprised the target expert panel. Responders were asked to select tests and metrics they believed were most relevant to informing physical readiness, injury risk mitigation, and successful course completion within the described military scenario:

“A physically arduous military training course has a high failure rate due to MSKI, voluntary withdrawal, and personnel not meeting the required standards during timed events. This multi-week selection course located in a mountainous region of the UK includes marching long distances (>25 miles) with heavy loads (>40 kg), and multiple timed best effort runs (orienteering) over shorter distances (3 to 10 miles) with reduced load carriage (10 to 25 kg). Technical and tactical activities occur during daytime and nighttime. Personnel are often sleep deprived and have restricted calorie intake. The Defense organization would like to understand whether collecting pre-course force plate data might inform future pass/failure rates and MSKI occurrence, and if so which force plate tests and metrics would responders recommend using and why?”

### 2.2 Selection of survey responders

Responders were purposively recruited based on their established publication record, applied experience, and/or recognized expertise in force plate testing and neuromuscular performance assessment. This included academics, applied sport scientists, strength and conditioning practitioners, and researchers with experience across tactical, athletic, and rehabilitation populations with experience using a variety of force plates across potential providers. To ensure diversity, we sought representation across professions, disciplines, countries, and perspectives (e.g., sport scientists, biomechanists and strength and conditioning specialists). Fifteen individuals were approached to respond to a survey based on their known experience and/or expertise in using force plate technology for the measurement of force production characteristics across a range of populations (tactical, elite sport and clinical). Previous exposure of working with military personnel was not essential, although evident in 60% of responders. Ten individuals responded to the survey and their responses were analyzed. The responder's expertise in using force plate technology across populations was considered crucial as most research and experience of force plate provision is coming from sectors outside of Defense (i.e., elite sport) and recommendations from a recent review (11) stating that gaining agreement towards standardizing force plate methodological practices should benefit from consensus involving both military and non-military practitioners.

### 2.3 Survey questions

Survey questions were developed by the authors (PL, NJR and PC) following completion of a recent scoping review (11) examining force plate methodological practices within military populations. A copy of the survey can be found in supplementary file 1. The survey design was additionally informed by current force plate literature, commonly reported force-time metrics, and practical considerations relevant to military screening environments. Responders were asked to consider 9 different types of force plate tests (reflecting all the types of force plate tests the authors are aware of) and answer a binary “*yes*” or “*no*” based on whether they believe the test is “important for informing performance characteristics prior to completing an arduous military training course”. The 9 types of tests included bodyweight tests, multi-joint isometric tests, single-joint isometric tests, CMJ, squat jump, drop jump, CMJ rebound, multi rebound jump tests and drop landing. Branching logic was used throughout the survey so that, if responders answered “*yes*” to any of these test categories, a comprehensive and standardized list of force plate-derived metrics was displayed. Responders were then asked to select the metric(s) they considered important to report from the standardized list. These metrics were broadly categorized (depending on the test type, for example isometric and dynamic activities) as “outcome-based”, “force-based”, “power-based”, “rate of force development (RFD)-based”, “impulse-based”, “velocity-based”, and “time-based” metrics. If they answered “*yes*” to any of these categories of metrics, a comprehensive and standardized list of force plate derived metrics would appear, and the responders were asked to select metrics from this list that they considered important to report. Responders were then asked to reflect on their responses (i.e., the metrics they selected from the dropdown list) and using freehand text list their personal “top 5” metrics for that specific force plate test. The option of listing metrics by freehand meant that alternative name/description for a force plate metric might be listed (for example, concentric and eccentric force, as opposed to propulsion and braking force). Other questions included: (1) *would you consider “loaded” force plate testing important to inform pre-course selection criteria?* and (2) *would you recommend the analysis and reporting of ratio-based metrics including “dynamic strength index” (DSI) or “eccentric utilization ratio” (EUR) prior to a selection course?*

### 2.4 Survey analysis

#### 2.4.1 Phase one. Establishing responder test and metric recommendations

Descriptive analysis of data was performed using Microsoft Excel. Consensus thresholds were selected *a priori* based on recommendations from Delphi methodology literature, where agreement thresholds between 51%–80% have commonly been used to indicate consensus, with 70% frequently adopted as representing strong agreement (40). Given the exploratory nature of the present study and the large number of potential force plate metrics available, a 70% threshold was selected for test inclusion and a 60% threshold for metric inclusion to avoid overly restrictive exclusion of potentially meaningful variables. Metrics selected with a frequency of 40%–50% were rated as having a “mixed response” and “potentially” worth considering within a future shortlist. A frequency analysis of responses to each question was performed to enable tests and metrics to be rated in order of importance. Summary tables and figures with the recommended shortlist of tests and metrics were created.

#### 2.4.2 Development of the final minimum dataset

The development of the final minimum dataset followed five sequential criteria. First, metrics meeting the prespecified survey threshold were retained for initial consideration. Second, metrics appearing in the responders' free-text “top five” selections on at least three occasions were also considered, including where variation in terminology may have dispersed responses across mechanically similar variables. Third, responder metrics were evaluated for biomechanical relevance to the construct represented by the test. Fourth, metrics were assessed for redundancy, including whether multiple variables expressed the same underlying force–time relationship or whether a ratio was uninterpretable without its component variables. Fifth, practical considerations, including; reliability, test duration, interpretability and feasibility within large military cohorts were considered. Each metric was then retained, reformulated or excluded.

The resulting minimum dataset was subsequently circulated to all survey responders and military specialists with UK Defence for comment on its technical coherence and practical feasibility. This final circulation was not treated as an additional anonymous Delphi round and did not alter the original survey frequencies. Rather, it served as a stakeholder review of the author-responses. The final recommendations should therefore be interpreted as expert-informed minimum dataset derived from survey findings and subsequent biomechanical underpinnings, rather than as a formal Delphi consensus statement.

## 3 Results

Table 1 provides a description of the ten responders who completed the survey. They predominantly include individuals who work as academics/researchers in the field of strength and conditioning who most frequently work with athletic populations.

**Table 1:** Survey responder demographics.

Responder demographics	Number
Profession	
Academic/Researcher in S & C	8
Researcher in both S & C and Physio	1
Applied Practitioner in S & C	1
Country of Employment	
Australia	3
United Kingdom	3
United States	2
Canada	1
Serbia	1
Experience working in Academia	
>10 years	7
7 to 9 years	1
4 to 6 years	1
<1 year	1
Experience as an applied practitioner	
>10 years	3
7 to 9 years	2
4 to 6 years	2
1 to 3 years	2
<1 year	1
Experience using force plate technology	
>10 years	6
7 to 9 years	2
4 to 6 years	1
1 to 3 years	1
Highest level of academic qualification	
PhD or Professional Doctorate	10
Populations worked with using force plate technology	
Elite/Professional Sport	8
Recreational Athletes	7
Tactical	6
General population	5
Clinical populations	2
Which force plate provider(s) do they primarily use?	
Hawkin Dynamics	8
Force Decks (Vald)	7
AMTI	3
Kistler	1
PASCO	1
Have you worked with military personnel?	
Yes	6
No	4

S & C, strength and conditioning.

### 3.1 Force plate “test” selection

From the nine different types of force plate tests included, only three tests met the threshold for being selected as an important test for inclusion as a pre-course assessment tool (each getting 9 “*yes*” responses out of 10). The 3 most selected tests include multi-joint isometrics; countermovement jump and drop jump. The force plate metrics selected for being important to report during these types of assessments are as follows:

### 3.2 Multi-joint isometric test “metric” selection

There was mixed consensus on the importance of collecting specific types of metrics for multi-joint isometric tests (e.g., the IMTP or isometric back squat) (Table 2). 90% of responders who recommend this type of test agreed that “force” based metrics were important. “Relative peak force” (*n* = 9) and “force at 100 ms” (*n* = 8) were most frequently reported as important metrics to collect when using multi-joint isometric tests. Other “force” based metrics had mixed agreement but met the thresholds for inclusion as a recommended test, they include “absolute peak force” (*n* = 5), “net peak force” (*n* = 5), “force at 200 ms” (*n* = 5) and “force at 150 ms” (*n* = 4), see Table 2. Whilst not included within the recommended Table 2, 30% of responders (*n* = 3) recommend reporting “limb symmetry [left/right (L/R) peak force %]” and “force at 250 ms” (see supplementary file 2).

**Table 2:** Force plate tests and metrics selection with strong and mixed agreement.

Metrics selected with strong agreement	Metrics with mixed agreement
Metrics selected during “*multi-joint isometric”* tests	
Relative Peak Force (N.kg^−1^) = 9	Absolute Peak Force (N) = 5
Force at 100 ms (N) = 8	Net Peak Force (N) = 5
	Force at 200 ms (N) = 5
	Time to peak force (s) = 5
	Force at 150 ms (N) = 4
	RFD 0–100 ms (N.s^−1^) = 4
Metrics selected during ‘*countermovement jump’* tests	
Metrics selected with strong agreement	Metrics with mixed agreement
Jump height (m) = 9	Propulsive phase duration (s) = 5
Take-off velocity (m.s^−1^) = 6	Average relative propulsive force (N.kg^−1^) = 4
Time to take-off (s) = 8	Average braking force (N) = 4
Braking phase duration (s) = 7	Average propulsive velocity (m.s^−1^) = 4
Countermovement depth (m) = 7	Peak braking velocity (m.s^−1^) = 4
Reactive strength index (modified) = 6	
Braking RFD (N.s^−1^) = 6	
Metrics selected during ‘*drop jump’* tests	
Reactive Strength Index = 9	Spring like correlation = 5
Jump Height (m) = 8	Landing Stiffness (N.m^−1^) = 5
Propulsive phase duration (s) = 7	Drop Height (m) = 4
Braking phase duration (s) = 6	Peak relative braking force (N.kg^−1^) = 4
	Peak propulsive force (N) = 4
	Average propulsive velocity (m.s^−1^) = 4
	Average braking velocity (m.s^−1^) = 4
	Time to peak braking force (s) = 4
	Countermovement depth (m) = 4

m, meters, s, second, m.s^−1^, meters per second, N, Newtons, W, Watts, kg, kilogram, RFD, rate of force development.

Four responders (out of 9) selected “yes” to the inclusion of “RFD” based metrics. All 4 responders consider “RFD 0–100 ms” important (which made the recommended shortlist, Table 2), 30% of responders selected “RFD 0–200 ms”, (failing to make the recommended shortlist, see supplementary file 2). Five responders (out of 9) selected “yes” to the inclusion of “impulse” based metrics. However, no impulse metrics made the recommended shortlist due to insufficient numbers agreeing on the most important impulse-based metric to report. Thirty percent of responders (*n* = 3) selected “impulse 0–100 ms” and “net impulse 0–100 ms” (failing to make the recommended shortlist, see supplementary file 2). Five responders selected “yes” to the inclusion of “time” based metrics and all five identified “time to peak force” as important metric to report and this made the recommended shortlist (Table 2).

When responders were asked to list their top 5 multi-joint isometric test metrics, 16 individual metrics were selected in total (Table 3). Only five of these metrics were listed by ≥3 responders, this included, relative peak force', “force at 100 ms”, “net peak force”, “time to peak force” and “impulse 0–200 ms”. It is important to note that responders were asked to provide their top 5 list using freehand. Similar metrics to the most frequently reported peak force values included “absolute peak force” (*n* = 2), “relative peak net force” (*n* = 2). Force at specific time points were commonly selected in responders “top 5” metric list (11 counts), but there was little agreement on the preferred time point. This led to only one of these specific metrics making the top 5 recommended list (force at 100 ms). Only one “impulse” metric made responders top 5 list and that was “impulse 0–200 ms” (*n* = 3). The recommended metrics to report for multi-joint isometric tasks when stratified into outcome and strategy-based metrics can be found in Table 4.

**Table 3:** List of metrics that made the responders top 5 shortlist.

IMTP	CMJ	Drop jump
Relative peak force (N.kg^−1^) = 6	Jump Height (m) = 7	RSI = 7
Force at 100 ms (N) = 5	RSI (modified) = 5	Jump height (m) = 5
Net Peak Force (N) = 4	Time to take-off/contraction time (s) = 4	Landing stiffness (N.m^−1^) = 3
Time to peak Force (s) = 4	Braking/eccentric RFD (N.s^−1^) = 3	Spring-like correlation = 3
Impulse 0–200 ms (N.s^−1^) = 3	Peak braking/eccentric force (N) = 3	Braking phase duration (s) = 3
Limb symmetry (L/R peak force) (%) = 3		Propulsive phase duration (s) = 3
Force at 200 ms (N) = 2	Peak propulsive/concentric force (N) = 2	Ground contact time (s) = 2
Force at 150 ms (N) = 2	Peak relative propulsive/concentric power (W.kg^−1^) = 2	Average propulsive/concentric power (W) = 1
RFD 0–100 ms (N.s^−1^) = 2	Countermovement depth (m) = 2	Braking/eccentric RFD (N.s^−1^) = 1
Absolute peak force (N) = 2	Jump momentum (Kg*m.s^−1^) = 2	L/R braking force (%) = 1
Force at 50 ms (N) = 1	Peak force (N) = 1	L/R propulsive force (%) = 1
Force at 300 ms (N) = 1	Average braking force (N) = 1	Peak braking force (N) = 1
RFD 0–200 ms (N.s^−1^) = 1	L/R peak braking force (%) = 1	Positive net impulse (N.s) = 1
RFD 0–250 ms (N.s^−1^) = 1	Braking/eccentric peak velocity (m.s^−1^) = 1	Average braking force (N) = 1
Net force/BM (N.kg^−1^) = 1	L/R average propulsive/concentric impulse index (%) = 1	Average propulsive force (N) = 1
Relative peak net force (N.kg^−1^) = 1	Relative propulsive net impulse (N.s/kg^−1^) = 1	Drop height (m) = 1
	Net Propulsive Power (W) = 1	Average braking net force (N) = 1
	Time to stabilization (s) = 1)	Countermovement depth (m) = 1
		Peak propulsive force (N) = 1
		Peak relative propulsive power (W.kg^−1^) = 1
		L/R Braking Impulse Index (%) = 1
		Time to Stabilization (s) = 1

The terminology used within Table 3 was provided free hand by the responders.

IMTP, Isometric midthigh pull, CMJ, countermovement jump, RSI, reactive strength index, L/R, Left/Right, m, meters, s, second, ms, meters per second, N, Newtons, W, Watts, kg, kilogram, RFD, rate of force development.

**Table 4:** Summary of “responder” recommended tests and metrics, stratified by “outcome” and “strategy” based metrics selection.

Outcome-based metrics	Strategy-based metric
Recommended metric selection for a multi-joint isometric test, such as the IMTP	
Relative Peak Force (N.kg^−1^)	Force at specific time-points (range 100 to 250 ms) (N)
Net peak force (N)	Time to peak force (s)
	RFD 0–100 ms (N)
	Impulse 0–200 ms (N.s^−1^)
	Limb symmetry (L/R peak force) (%)
Recommended metric selection for the CMJ	
Jump height (m)	Braking phase duration (s)
Reactive strength index (modified)	Propulsive phase duration (s)
Take-off velocity (m·s^−1^)	Countermovement depth (m)
	Average relative propulsive force (N.kg^−1^)
	Average braking force (N)
	Peak braking force (N)
	Time to take-off (s)
	Braking RFD (N.s^−1^)
	Average propulsive velocity (m.s^−1^)
	Peak braking velocity (m·s^−1^)
Recommended metric selection for the drop jump	
Reactive Strength Index	Propulsive phase duration (s)
Jump height (m)	Braking phase duration (s)
Drop height (m)	Peak relative braking force (N.kg ^−1^)
	Peak propulsive force (N)
	Average propulsive velocity (m·s^−1^)
	Spring like correlation
	Landing Stiffness (N.m^−1^)
	Time to peak braking force (s)
	Countermovement depth (m)

IMTP, Isometric midthigh pull; CMJ, countermovement jump; RFD, rate of force development; m, meter; s, second; m·s^−1^, meters per second; N, Newtons; W, Watts; kg, kilogram.

### 3.3 Countermovement jumps “metrics” selection

The recommended metrics to report for the CMJ can be found in Table 2. Based on the outcomes of the survey, responders did not agree on the most important metrics for the CMJ test. All 9 responders who recommend this type of test agreed that “outcome”, “force” and “time” based metrics were important. Between 50% and 60% of responders felt it important to collect “power”, “impulse”, “RFD” and “velocity” based metrics.

There was strong consensus on the need to collect the following “outcome” based metrics, “jump height”, “take-off velocity” and “reactive strength index (RSI) (modified)”. Whilst there was wide consensus on the need to collect “force” based metrics, there was reduced agreement on which specific “force” based metrics are important to report. Subsequently only two force-based metrics made the recommended shortlist including “average relative propulsion force” and “average braking force”, which were both selected by 4 responders (Table 2). Thirty percent of responders selected “peak relative propulsive force”, “peak braking force”, “peak relative landing force”, “average propulsive force” and “peak relative propulsive force” but these failed to meet the minimum threshold for inclusion as a recommended CMJ metric (see supplementary file 2). There was strong agreement on the need to report “time” based metrics (*n* = 9) with three “time” metrics making the recommended short list, including “time to take-off”, “braking phase duration” and “propulsive phase duration” (see Table 2). Thirty percent of responders recommended “time to peak force” and “propulsive force duration” but these failed to meet the recommended shortlist.

Five responders (out of 9) selected “*yes*” to the inclusion of “power” or “impulse” based metrics. However, no “power” or “impulse” metrics made the recommended shortlist due to insufficient numbers (3 responders or less) agreeing on the most important metrics to report. Only one metric was recommended by 30% of responders and this was “impulse ratio” (see supplementary file 2). Six responders (out of 9) felt it important to collect “velocity” based metrics, with two metrics having a mixed consensus but making the recommended list, they include “average propulsive velocity” and “peak braking velocity”. Six responders (out of 9) felt it important to collect “RFD” based metrics, with one metric having strong agreement (braking RFD). Thirty percent of responders thought “L/R average braking RFD” should be reported, but this was not shortlisted for recommendation (see supplementary file 2).

When responders were asked to list their “top 5” CMJ test metrics, 17 different metrics were selected in total, however only five metrics were selected by ≥3 responders. These metrics included, “jump height”, “RSI (modified)”, “time to take-off/contraction time”, “braking RFD” and “peak braking force” (see Table 3).

### 3.4 Drop jump “metrics” selection

The recommended metrics to report for the drop jump can be found in Table 2. There was mixed consensus on the importance of collecting specific types of metrics for the drop jump test. All 9 responders who recommend a drop jump test agreed that “outcome”, and “time” based metrics were important to report, with 70% responders recommending “force” based metrics. Five responders felt it important to collect “impulse” and “velocity” based metrics. Only three responders recommended the collection of “RFD” based metrics and two responders recommended collecting “power” based metrics (see supplementary file 2).

There was strong consensus on the need to collect “outcome” based metrics, with “jump height” (80%) and “RSI” (90%) both being highly recommended. Whilst there was a consensus on the need to collect “force” based metrics, there was reduced agreement regarding the most important “force” based metric(s) to report. Subsequently only two “force” based metrics made the recommended shortlist including “peak relative braking force”, and “peak propulsive force”, both selected by 4 responders (Table 2). Thirty percent of responders selected “L/R peak braking force”, “average braking force”, “average propulsive force”, “L/R average propulsive force” and “L/R propulsive force”, but these were not selected frequently enough to meet the threshold for recommendation (see supplementary file 2). There was strong agreement on the need to report “time” based metrics (*n* = 9) with three “time” metrics making the recommended short list (“propulsive phase duration”, “braking phase duration” and “time to peak braking force”, see Table 2). Thirty percent of responders also recommended “time to stabilization” but this was not shortlisted (see supplementary file 2).

Five responders (out of 9) selected “*yes*” to the inclusion of “impulse” based metrics. However, no “impulse” metrics made the recommended shortlist due to an insufficient number of responders (3 responders or less) agreeing on which impulse metric is most important to report. Thirty percent of responders selected “impulse ratio” and “L/R braking impulse”, but these did not make the shortlist (see supplementary file 2). Five responders (out of 9) felt it important to collect “velocity” based metrics, with two velocity metrics making the recommended list, they include “average propulsive velocity” and “average braking velocity”. Metrics labelled under the descriptor “other” which made the recommended shortlist include 'spring like correlation', “landing stiffness” and “countermovement depth” (Table 2). Using box heights between 30 and 40 cm was considered most important when administering a drop jump task in this military context.

When responders were asked to list their “top 5” drop jump test metrics, 22 individual metrics were selected in total. Six of these metrics were listed by ≥3 responders, these included, “RSI”, “jump height”, “landing stiffness”, 'spring like correlation', “braking phase duration” and “propulsive phase duration”. “Contact time” only featured in the top 5 metrics of 2 responders (Table 3).

### 3.5 Other considerations

When responders were asked whether they considered “loaded” force plate testing important to inform pre-course selection criteria, 60% reported “*yes*” and 40% reported “*no*”. In the 6 responders who selected yes to this question, 5 recommended loaded CMJ and 4 recommended a loaded squat jump as the preferred test. When asked about the usefulness/importance of reporting ratio-based metrics to inform pre-course selection (50%) recommend reporting DSI and (50%) recommend reporting EUR.

## 4 Discussion

This is the first study which aimed to gain a consensus on which force plate tests and metrics should be selected by military training staff to better screen physical performance characteristics prior to service personnel attending physically arduous military training/selection courses. The results of this practitioner survey have revealed consensus among experienced force plate practitioners on force plate “test” selection but mixed consensus when it comes to recommended “metric” selection among experienced force plate users.

### 4.1 Force plate test selection

Multi-joint isometric (e.g., IMTP), CMJ and drop jump were the three force plate tests recommended to inform the physical readiness of service personnel prior to a physically arduous military selection course. The broad consensus of these three tests being most useful to inform military physical preparation and injury mitigation strategies is logical. When used in combination, these three tests would enable the determination of maximal and rapid force production characteristics [maximum isometric force production and RFD (i.e., IMTP); ballistic, slow SSC force production (i.e., CMJ); and reactive, fast SSC force production (i.e., drop jump)] (41). The assessment of personnel across such force production characteristics (i.e., maximal isometric strength, slow SSC and fast SSC function) would provide a comprehensive needs analysis of athletic performance profiling (3, 6, 7). Whilst consensus on force plate “test” selection was clear, the next most important consideration is metric selection. Responses to this survey reveal mixed opinions on which metrics are most important for informing pre-course physical preparation strategies. This highlights the fact that greater research is required to determine the physical qualities, associated tests, metrics and performance thresholds which increase the likelihood of success during arduous physical training courses, such as special forces selection.

### 4.2 Multi-joint isometrics “metric” selection

There is clear consensus that reporting expressions of peak force and force at specific time points are important. However, there was not wide held agreement on the specific time points of central interest, with force at 100 ms, 150 ms and 200 ms all being shortlisted for recommendation and force at 50 ms (*n* = 2), 250 ms (*n* = 3) and 300 ms (*n* = 2) all being acknowledged for their potential value to the end user. As the time point increases, reliability of measurement also improves (24, 42, 43), an important factor when identifying metrics to inform applied practice. In addition, many MSKI (e.g., non-contact anterior cruciate ligament and ankle injuries) are reported to occur in <100 ms (44–47), as such, assessing force production capability over shorter time frames may be informative of potential injury risk and during injury rehabilitation, thus justifying the reporting of force at a range of time points during isometric testing.

Absolute force is reported either as “gross” (i.e., including body mass) or “net” (i.e., excluding body mass). Despite net force being utilized in the early IMTP studies (48–50) and more recently used to identify changes in neuromuscular function during special forces selection (51), specifying which of these metrics (“gross” or “net” force) is essential, but unfortunately uncommon in previous military literature (11). To fully exclude the influence of stature/mass on performance, relative net force should be reported. For some military tasks (e.g., manual material handling, casualty evacuation) a minimum absolute threshold may be required and therefore net peak force may be of importance.

RFD 0–100 ms was the only RFD metric shortlisted (by 4 responders). Reliability concerns are raised when reporting RFD metrics from multi-joint isometric tests, particularly with early epochs (42). When RFD has been used, many researchers do not clearly report their methods of calculation (11) for example, peak or mean RFD, or a moving average window which has been shown to notably affect the magnitude and reliability of the values (42). It may be that RFD over 0–100 ms was selected due to the short epochs over which injuries are reported to occur (44–47).

**Figure 1:**
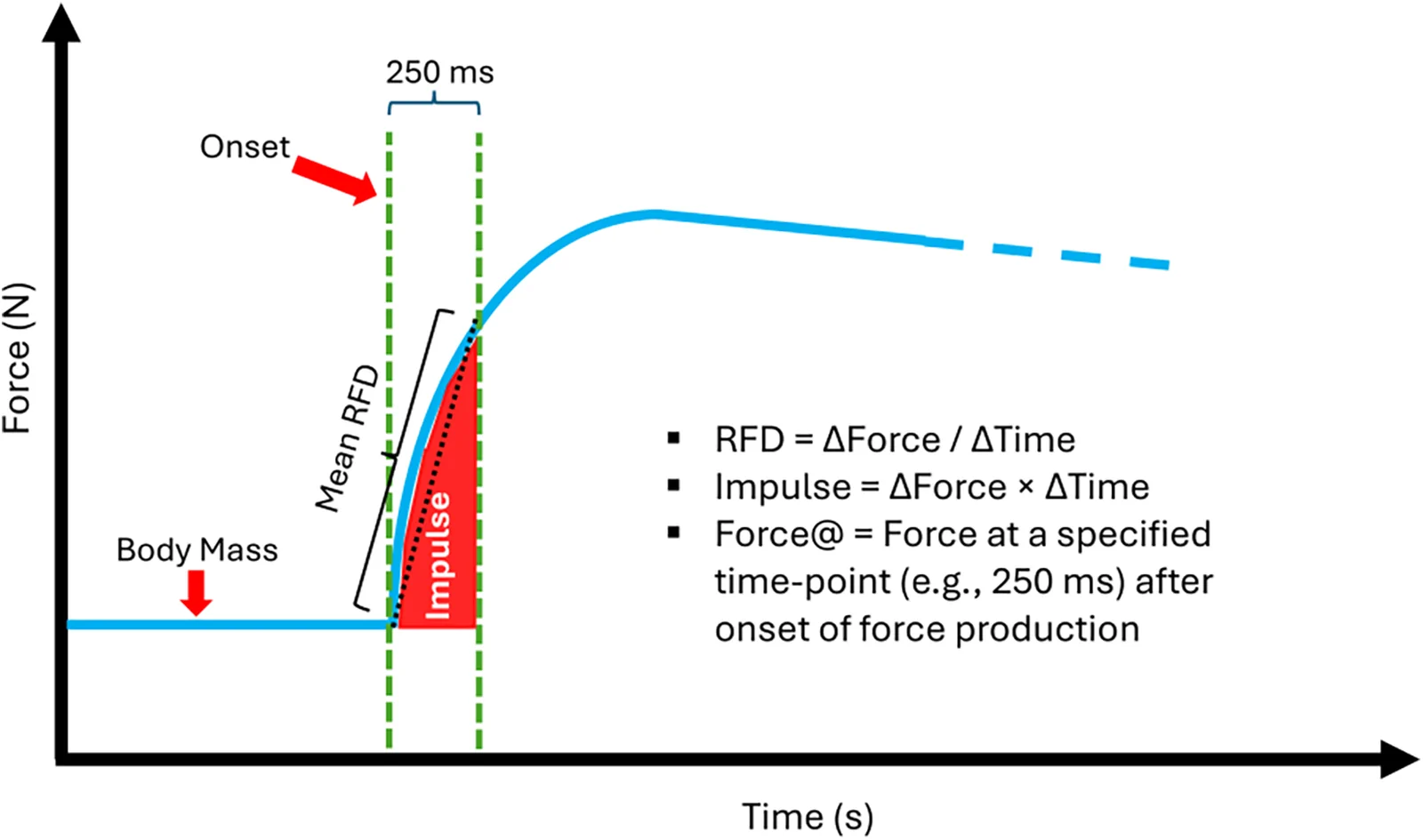
Representative force trace from an isometric mid-thigh pull. Illustrating how mean rate of force development (RFD) 0–250 ms, impulse 0–250 ms and force at 250 ms effectively provide the same information. If force at 250 ms changes in response to training, both RFD 0–250 ms and impulse 0–250 ms will change to the same magnitude, during an isometric assessment.

Thirty percent of responders identified “impulse 0–200” and limb symmetry (“L/R peak force symmetry %”) within their top 5 metrics for the IMTP meaning that these metrics made the recommended shortlist. It is important to note that not all of the temporal metrics are required, for example, if force at specific time-points is reported then RFD and impulse (and time to peak force due to collinearity with force at specific time-points) are redundant. During longitudinal monitoring, if force at 250 ms changes, RFD over 0–250 ms and impulse over 0–250 ms would change by a similar magnitude as the time period is constant (Figure 1). Force at specific time-points may also introduce less variability than metrics such as RFD, due to the fact researchers have used multiple methods of calculating RFD (e.g., peak, average and the use of moving average windows) (42). However, it is imperative that data processing methods are considered, clearly reported in the research and standardized, as different onset thresholds notably affect time related metrics (24, 52, 53).

Reporting limb symmetry during multi-joint isometric testing was recommended by some responders. However, it is also important to consider the component parts (i.e., force produced), not just asymmetry (reported as % difference) (54) because muscle strength is an important predictor of non-contact MSKI (19–21) and therefore greater relative peak force would likely offset risk of MSKI (55). For example, if a service person, can produce 50 N•kg^−1^ but have a 15% asymmetry, and a different service person can produce 25 N•kg^−1^, but is perfectly symmetrical, it is likely that the weaker person is the one at risk of a MSKI, as both legs are relatively weak, although additional research is needed to confirm this. When calculating asymmetries, it is also important to consider that asymmetries may vary depending on the metric selected to calculate the asymmetry as has been shown for the CMJ (56).

### 4.3 Countermovement jumps metrics

Seven metrics were selected that shared strong agreement among responders (see Table 2). Nine responders agreed on the requirement to collect some form of “force” based metrics, however, there was little agreement on which specific “force” based metrics should be selected for reporting purposes. This lack of consensus was due to a difference of opinion regarding the reporting of “average” force values vs. “peak” force values, and the jump phase (“propulsive” force vs. “braking” force values). “Peak” is the highest force recorded over one millisecond, and does not impact jump height, whereas “mean” propulsive force is a component of “relative net propulsive impulse” which does influence jump height (17, 57). Subsequently, mean force values (not peak) should be considered when selecting CMJ metrics, which should be identified during the braking or propulsive phases.

“Power”, “impulse” and “velocity” based metrics were considered important to report by 50 to 60% of responders. However, a lack of agreement on which specific “power”, “impulse” and “velocity” metrics should be reported were demonstrated due to nuances relating to the phase of the jump (i.e., braking or propulsive), whether to report “peak” vs. “mean” values, and “gross” vs. “net” vs. relative [scaled by body mass] values). Because of this, the highest scoring of these metrics (peak braking velocity) was only selected on 4 occasions. However, peak braking velocity, would occur at the onset of braking, prior to deceleration occurring, and therefore only providing insight into the initial unweighting/descent phase. Interestingly, Linthorne et al. (17) and McBride et al. (57) explain how power metrics are not important as they do not explain jump height; it is “relative net propulsive impulse” that determines velocity at take-off and take-off velocity determines jump height (a higher jump height requires a higher velocity at take-off). As such, to gain insights into slow SSC function, relative net mean propulsive force and propulsive phase duration should be used as strategy-based metrics to provide insight into how the jump height was achieved (e.g., a high force over a short duration, or a lower force over a longer duration), potentially informing future training prescription.

### 4.4 Drop jump metrics

Only 20% of responders reported contact time in their “top 5” metric shortlist, even though a criterion of <250 ms ground contact time should be applied for an acceptable trial (58–60) and 2 responders were happy to report RSI without the components (Table 3). However, reporting ratio metrics, such as RSI, without the components of the ratio (i.e., jump height and ground contact time) only provide part of the picture, as it is possible to achieve the same RSI but with completely different strategies (for example, service personnel 1–3 in Table 5). Alternatively, the same jump height with different ground contact time will result in notably different RSI scores (for example, service personnel 1, 4 & 5 in Table 5). In addition, if the best performance is taken based on RSI, service person 5 would be considered the best performer, although if jump height was used, service person 3 would be the best performer, and if ground contact time was used service persons 2 and 5 would be comparable (Table 5). These examples highlight why it is important to consider both the jump height and ground contact time.

**Table 5:** Interaction between jump height and ground contact time when calculating reactive strength index.

Metric	SP 1	SP 2	SP 3	SP 4	SP 5
RSI (AU)	2.0	2.0	2.0	2.22	2.67
Jump height (m)	0.40	0.30	0.50	0.40	0.40
GCT (s)	0.20	0.15	0.25	0.18	0.15

SP, service person; RSI, reactive strength index; AU, arbitrary units; m, meters; GCT, ground contact time; s, seconds.

No “power”, “impulse” or “RFD” based metrics were shortlisted when reporting a drop jump. This should be unsurprising because power and RFD do not determine jump height. Braking impulse would inform us of their fall height, based on the impulse momentum relationship (17, 57). Propulsive impulse, if net and relative to body mass explains jump height, so jump height and the duration of each phase may be sufficient to report, although relative mean force during each phase would provide complete insight.

Not all responders considered drop height necessary to effectively interpret drop jump data. Of course, drop height would need to be known to accurately calculate many drop jump metrics, but it may not need to be reported. Performance of the drop jump relies on the assumption that individual(s) fall from the height of the box or elevated platform. However, the difference between actual fall height and box heights range between 28.6–39.5% in both directions either lowering or raising the center of mass prior to initiating the drop (i.e., 0.30 m box height equals an actual fall height between 0.18–0.42 m) (61, 62). This discrepancy between box height and fall height alters the mechanical demands of any drop jump assessment compromising data accuracy of the drop jump. This collectively highlights the importance of monitoring actual drop height when screening physical performance characteristics. This omission could be related to this metric not being an available optional on all force plate providers software. However, there is now a method of calculating fall height, rather than assuming fall height and box height are the same, which may resolve this problem (63). Although to our knowledge this is only currently built into two of the commercially available automated force plate software systems. Not negating access to this specific metric from a chosen force plate software, describing the box height used when completing a drop jump is however, an essential reporting metric when using a drop jump test.

### 4.5 Other considerations

There was a lack of consensus for the use of reporting ratio-based metrics such as DSI and performing “loaded” force plate tests to inform physical preparations and injury mitigation prior to attending a military selection course. As with other ratio metrics, such as RSI, it is important to know the components of the ratio, as a high DSI can be achieved if made up of a low CMJ peak force and a low IMTP peak force (e.g., CMJ peak force: 1,998 N/IMTP peak force: 2,231 N = 0.90) clearly indicating that the individual needs to emphasize strength development, while if CMJ peak force was lower (e.g., CMJ peak force: 1,225 N/IMTP peak force: 2,231 N = 0.55), resulting in a reduced ratio, this may indicate from the DSI that individual needs to emphasize ballistic training, even though they are weak. If both examples had a body mass of 80 kg this would result in a relative peak force of 27.9 N•kg^−1^, also indicating that strength development should be emphasized, irrespective of the DSI ratio. DSI can be beneficial in determining if an individual has adapted to a block of training in the manner expected and in combination with consideration of their relative strength levels, this information can be used to determine the training priorities for the next phase of physical training (64–67), however, DSI alone may provide limited insight regarding readiness for arduous training.

It is somewhat surprising that loaded jump testing was not suggested by the responders, considering the load carriage requirements in the military, especially during some of the more strenuous training courses. This may be due to limited published research using such an approach in military populations. Alternatively, this could be due to the fact that both jumping and landing strategy appear to change as load increases, with a progressive, although not linear decrease in jump height and an increase in propulsive duration, while there is an initial increase in landing impulse followed by a decrease in landing impulse at higher loads, due to an interaction between decreased jump height and increase in landing duration (68, 69).

### 4.6 Top 5 metric shortlist

When responders were asked to provide their top 5 most important metrics against each selected test there were wide inconsistencies in reporting. For the CMJ and drop jump test there was little agreement on 'strategy' based metrics, only agreement on “outcome” based metrics. This suggests that even among experienced force plate practitioners there are mixed perspectives when it comes to reporting practices for dynamic force plate tasks for informing occupational task performance. Developing more research and achieving consensus towards standardizing these reporting practices are needed. Well-established criterion data analysis procedures do exist (70, 71), however, not all software packages adhere to these data analysis procedures, making some comparisons between systems problematic, particularly for dynamic tests (70, 71). For example, the recommended threshold for the identification of the onset of movement during a CMJ is a decrease in force which exceeds 5 standard deviations of the force measured during a 1 s period of quiet standing (72) with a backward search to start integration of the data 30 ms prior to this decreases in force to find the last force equal to body weight (72). However, this is not the default threshold in software systems and such differences in onset thresholds (and jump phase identification) can affect temporal variables and the calculation of velocity (70, 71), and as such when comparing data to published data, careful consideration of not only the testing methodologies, but also the data analysis procedures are essential (11, 73).

### 4.7 Recommended force plate test and metric selection vs. historically reporting in military personnel

Recently authors of a scoping review (11) described the methodological reporting practices of force plate derived measure force production characteristics in military personnel. The review included 40 articles and provides a nice synopsis of how force plate tests and metrics derived from the IMTP and CMJ are currently being used across military settings.

Of the 10 articles that included the IMTP in this review (11), half (*n* = 5) reported one metric only (25, 33, 73–76), 4 articles (9, 77–79) reported 2 or 3 metrics, and one article (31) reported 4 metrics. Eight of these articles (80%) relied entirely on “outcome” based metrics (i.e., peak force). Two articles (31, 79) included RFD but over different epochs (0–100 and 0–250 ms). Limb symmetry (%) and time-to-peak force was recorded in one article only (31). Force at specific time points were commonly recommended by responders to this survey, however no articles included in the review by Ladlow et al. (11) used any of these strategy-based metrics when reporting IMTP performance. This observation demonstrates a historical underutilization of strategy-based metrics when using the IMTP.

Twenty-eight of the 40 articles included within this same review (11) reported the CMJ. Most articles reported one or two metrics only (*n* = 17, 61%). Three articles (11%) reported between 3 and 4 metrics, 6 articles (21%) reported 5 to 7 metrics. The two articles with the most comprehensive force-time analysis of CMJ included Merrigan et al. (80) (*n* = 9 metrics) and Bird et al. (28) (*n* = 14 metrics). This also demonstrates a historical underutilization of strategy-based metrics when using the CMJ. Two of the most reported metrics from this review (11) include expressions of peak power and peak force. Thirteen articles reported “power” based metrics [peak power [*n* = 9], relative peak power [*n* = 6], average power [*n* = 3]] and 6 articles reported different expressions of force [peak force [*n* = 3, 11%], relative peak force [*n* = 2, 7%], average force [*n* = 2, 7%]]. However, neither of these metrics determine jump height (16, 17, 56) and their inclusion for informing pre-course selection physical preparedness should be questioned. As an example, the correlation between jump height and peak power is artificially inflated by the near-perfect correlation between jump height and the velocity at peak power (17). Also, power can increase while jump height decreases and vice versa, based on changes in jump strategy. Interestingly, RSI (modified), a metric with strong responder agreement as a recommended metric within this survey was only reported in 3 (11%) (28, 80, 81) of the 28 articles that measured CMJ (11). Collectively, this demonstrates a historical underutilization of both outcome and strategy-based metrics for the CMJ.

Power remains a widely used metric in CMJ literature, whilst we stand by the biomechanical rationale for its exclusion in our final recommendations, if power is being evaluated, its inclusion should be justified, it should not be used in isolation and include its component parts (work and time). Inclusion of the component parts permits the determination of any changes in strategy even if power does not change, similar to considering net propulsive force and propulsion phase duration when jump height has not changed.

Two examples of the drop jump test being used in military performance settings include articles by Debenedictis et al. (82) and Merrigan et al. (83). Using a box height of 0.65 m, Debenedictis et al. (82) selected 3 metrics; (1) “peak landing force”, (2) “time to peak landing” and (3) “rate of force production”. The coaching cues included “minimizing ground contact time” before performing a maximal vertical jump. The other drop jump article by Merrigan et al. (83) selected 9 metrics to be reported under three different drop jump conditions [30 cm loaded (15 kg), 30 cm unloaded and 60 cm unloaded]. These metrics included “contact time”, “jump height”, “RSI”, “peak eccentric force”, “peak concentric force”, “eccentric impulse”, “concentric impulse”, “eccentric RFD” and “time to peak eccentric force”. The mean ground contact times reported from this study ranged between 0.59 s and 0.69 s (83). In both articles (82, 83) authors did not control for ground contact times being <250 ms when administering a drop jump task, meaning that fast SSC function was not being assessed as the strategy was compliant (based on the duration of ground contact) making these tasks depth jumps (i.e., slow SSC task) rather than drop jumps. Not controlling for fast ground contact times (i.e., <250 ms) within a drop jump alters both the mechanical output and structural behavior within the task (84–86). Specifically, longer ground contact and resultant larger angular displacement results in changes to contractile and elastic tissue function during the task, i.e., the structures are more compliant (84–86). Across the literature there is common misnomer for drop jumps exceeding 250 ms, where they should be more accurately defined as depth jumps and they should not be used interchangeably (87). Interestingly, Debenedictis et al. (82) did not report the majority of metrics recommended by the responders to the survey, whereas the Merrigan et al. (83) did report many of the metrics shortlisted by responders.

It is important to mention the recent publication of an article by Uphill et al. (51). This article investigated changes in physical performance in response to the Australian Special Forces selection course and monitored recovery throughout 8 weeks following the course. They incorporated the IMTP and CMJ test into their physical performance battery. The authors reported nine IMTP metrics, including unfiltered “peak force”, “relative peak force”, “force at 100, 200, and 250 ms”, “peak RFD” and “RFD at time bands 0–100, 0–200, and 0–250 ms” all based off net force. They also selected eight CMJ metrics, including “jump height” from take-off velocity, “propulsive peak force”, “propulsive impulse”, “peak power”, “mean power”, “braking RFD”, “braking peak force”, and the “ratio of flight time to contraction time”. To our knowledge, this article offers the most comprehensive analysis of both outcome and strategy-based metrics that most closely align with the metric recommendations listed by responders to this survey and are also pertinent to informing physical readiness prior to a physically arduous military selection course.

### 4.8 Final metric recommendation

**Figure 2:**
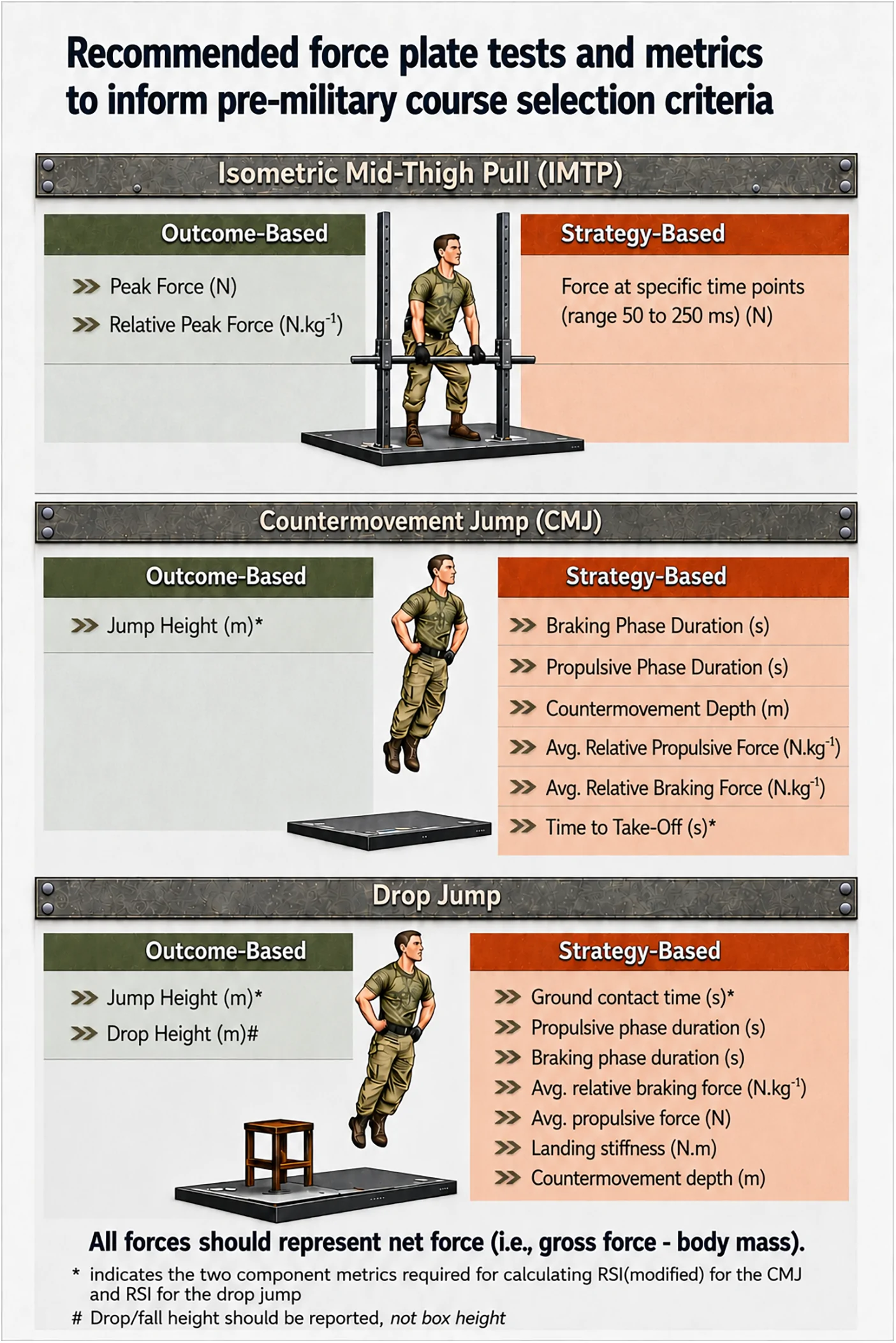
Expert-informed minimum force plate dataset proposed to complement physical-readiness assessment before physically arduous military training. Note. for illustration purposes only, the trunk angle during the IMTP should be more upright with no more than a 10 degree forward lean. Image generated using ChatGPT.

Lack of agreement or consensus among responders often stems from which phase of the jump should be reported, peak vs. mean calculations, and gross vs. relative vs. net values. In previous sections of this article, the authors have provided a rationale for the inclusion and removal of some force, power, RFD and impulse based metrics recommended by responders (Figure 1). Following further scrutiny of the responder-created recommended “outcome” and 'strategy' based force plate metrics listed for the multi-joint isometric test (e.g., IMTP), CMJ, and drop jump test (Table 4) the authorship provides what they consider a minimum dataset of metrics that they feel should be incorporated into the pre-course assessment battery (Table 6 and Figure 2). While some of these recommendations do not agree with previous recommendations for the CMJ (38), the redundancy of many of the metrics [e.g., peak power and therefore time to peak power; RSI (modified) without the component parts] recommended by Bishop et al. (38) have already been explained, based on underpinning Newtonian principles.

**Table 6:** Minimum list of recommended metrics to be reported as part of a pre-course screening.

Outcome-based metrics	Strategy-based metric
Recommended metric selection for a multi-joint isometric test, such as the IMTP	
Net peak force (N)	Force at specific time points (range 50 to 250 ms) (N)
Relative peak force (N.kg^−1^)	
Recommended metric selection for the CMJ	
Jump height (m) *	Braking phase duration (s)
	Propulsive phase duration (s)
	Countermovement depth (m)
	Average relative propulsive force (N.kg^−1^)
	Average relative braking force (N.kg^−1^)
	Time to take-off (s)*
Recommended metric selection for the drop jump	
Jump height (m)*	Propulsive phase duration (s)
Drop height (m)#	Braking phase duration (s)
	Average relative braking force (N.kg^−1^)
	Average propulsive force (N)
	Landing Stiffness (N.m^−1^)
	Countermovement depth (m)
	Ground contact time (s)*

* Indicate the two component parts required to calculate RSI (modified) for the CMJ and RSI for the drop jump.

# Drop/fall height should be recorded, not box height.

IMTP, Isometric midthigh pull; CMJ, countermovement jump; RFD, rate of force development; m, meter; s, second; N, Newtons; W, Watts; kg, kilogram.

### 4.9 Research and applied implications

When performing data collection with large numbers of service personnel, selecting tests that are quick to administer becomes a logistical priority. Participant numbers in the two drop jump test studies detailed in this article are quite small (*n* = 16) (82) and (*n* = 26) (83). For perspective, 16 articles included in the scoping review (11) recruited over 100 personnel, 14 of these articles used the CMJ as the primary force plate test to measure rapid force production characteristics. Among the challenges of incorporating the drop jump into a pre-screening physical performance battery is the coaching time burden to enable, often inexperienced and mixed ability groups, the opportunity to execute a 'successful' test. For example, stepping off the box at a specific height, as opposed to “jumping” off or “dropping” off the box, and producing a ground contact time < 250 ms, for a minimum of 3 successful attempts). This “time” component needs to be considered if using the drop jump alongside the IMTP and/or CMJ in future pre-course selection physical performance batteries. Rapid force production could also be assessed using a shortened IMTP protocol, if time permits, which enhances the reliability of the metrics (24, 88).

#### 4.9.1 Future validation of the recommended minimum dataset

The final recommendations identify a minimum dataset; it does not establish predictive validity or criterion standards. Prospective studies are required to determine whether these metrics are associated with MSKI, voluntary withdrawal, course completion and occupational performance during physically arduous military training. Such studies should include established predictors; including aerobic fitness, maximal strength, muscular endurance, load-carriage performance, demographic characteristics, prior military role and previous course experience to determine whether force plate measures provide incremental explanatory or predictive value.

Validation should occur in sequential stages. First, reliability and measurement error should be established under the exact protocols and processing methods intended for military use. Second, longitudinal studies should determine responsiveness to physical preparation, cumulative fatigue, sleep restriction, energy deficit and recovery. Third, multivariable prediction models should evaluate whether baseline or change scores improve discrimination and calibration beyond conventional assessments. Fourth, any proposed criterion thresholds should be derived in adequately powered cohorts and externally validated in independent military populations before being used for candidate selection or risk stratification.

The practical value of the proposed battery will depend on incremental rather than isolated validity. A force plate metric need not outperform aerobic fitness or load-carriage performance to be useful, but it should provide non-redundant information that changes interpretation, training prescription or longitudinal monitoring. Future studies should therefore compare models containing conventional predictors with and without force plate variables rather than evaluating force plate metrics in isolation.

It will also be important to determine whether a smaller subset of metrics provides equivalent practical information to the full recommended list. Until this work is completed, the proposed dataset should be used for standardised profiling and research rather than as a stand-alone pass/fail instrument.

## 5 Limitations

We acknowledge that the responder sample was relatively small. However, expert consensus methodologies commonly prioritize participant expertise and purposive recruitment over sample size alone, particularly within specialized domains where the pool of appropriately experienced individuals is limited (40, 89). Similar expert-led recommendation papers within sports science, biomechanics, and force plate assessment have used comparably sized expert groups when developing applied frameworks and metric-selection guidance (38, 60). Among the strengths of the present study is that we analyzed the responder feedback and then shared the findings with military specialists to ensure that the final recommendations are implementable in practice. While a consensus was not clear from the initial survey responses, all authors came to a consensus by the end of the development of the manuscript. Nevertheless, additional research involving larger international tactical expert panels may strengthen future recommendations.

Although the survey was grounded in a preceding scoping review (11) and underwent two rounds of internal pre-testing, it did not undergo an independent formal content-validity exercise. It therefore remains possible that alternative wording, categorisation or metric nomenclature might have influenced some metrics selections. Each respondent represents 10% of the sample, distinctions between “strong agreement” and “mixed agreement” among responder recommendations may be unstable. For example, a difference between 40% and 50% agreement reflects only one respondent. This reflects the limited resolution of percentage-based interpretation in such a small sample.

Although responders demonstrated agreement for several tests and metrics, the survey design did not formally explore the reasoning underpinning each decision. Future qualitative work may help identify why practitioners prioritize specific metrics.

## 6 Practical applications

Defense organizations are continually looking at methods to maximize the numbers of military personnel available to support the main objectives of elite military units. Understanding which metrics are important to measure, identifying training deficits and future revisions to exercise prescription and program design prior to course can only be effectively undertaken based on the selection of valid, reliable and sensitive metrics from each force plate test. This facilitates the accurate interpretation of both maximal and rapid force production characteristics that are essential to ensure training/operating staff have increased confidence in their physical preparation decision-making criteria (i.e., who is physically prepared to attend the next selection course). Where testing time is constrained, practitioners should first consider whether the resulting data will answer a predefined decision or monitoring question. A smaller number of reliable and interpretable metrics is preferable to indiscriminate extraction of all variables available within commercial software. The minimum dataset proposed here provides a standardised starting point for that process and for future research evaluating whether force plate assessment adds value beyond conventional military testing.

The final recommendations describe an expert-informed minimum dataset for characterising maximal isometric, slow SSC and fast SCC force-production characteristics. It is intended to complement established military assessments such as aerobic fitness, maximal strength, muscular endurance, load carriage and occupational task performance and should be administered sufficiently in advance of course attendance to inform profiling and, where appropriate, physical preparation.

Currently, the principal defensible application for this recommended test and metric dataset include standardised neuromuscular profiling, longitudinal monitoring and evaluation of physical-preparation interventions. Such information may help practitioners formulate and evaluate individualised training hypotheses. The recommendations do not currently provide evidence-based pass/fail thresholds, minimum course-entry standards or individual injury-risk classifications. Before force plate outcomes can inform candidate-selection decisions, prospective studies must determine whether they explain meaningful additional variance beyond established measures such as aerobic fitness, maximal strength, muscular endurance, load-carriage performance, prior military experience and occupational task performance. Until that evidence is available, force plate results should not be used in isolation to exclude personnel from training or to infer individual injury risk.

No universal percentage deficit can presently be translated into a prescribed duration of additional preparation. However, one example of how the recommendation framework could be applied in the future include; (1) identifying service personnel who are physically prepared to attend the next available course intake, (2) identify candidates nearly at the standard and subsequently might benefit from a small revision to their training program (for example, a candidate scores less than a 10% deficit in an important metric that could be met with 3 months of targeted exercise training), and (3) identify service personnel in need of a lot more targeted maximum strength or velocity-based training (or both) (for example, if a candidate demonstrated greater than 25% deficit in an important metric, they should probably not be invited onto the next course intake but instead be encouraged to invest 6 to 12 months training specific deficits so they are better prepared for future course intakes). Criterion values will require prospective derivation and external validation against clearly defined military outcomes before this can be implemented into practice.

## 7 Conclusion

Researchers and applied practitioners wishing to use force plate technology should be well placed to identify the most important tests and metrics to answer their specific research question(s). However, due to time and logistical challenges, practitioners must be selective when designing their physical performance screening battery, often delivered to large military cohorts. Experienced force plate users prioritised multi-joint isometric, CMJ and drop-jump testing as primary force plate methods for complementing physical-preparation assessment before physically arduous military training courses. The final recommendations represent an expert-informed, biomechanically reasoned minimum force plate dataset intended to complement established military physical-readiness assessment and standardise future prospective research.

The final recommendations (Table 6/Figure 2) offers training/operating teams guidance beyond reporting “outcome” based metrics alone and could be considered a minimal template of metrics for practitioners to use when screening/profiling service personnel prior to attending physically arduous military courses (such as elite infantry unit selection).

The proposed minimum dataset should facilitate more consistent neuromuscular profiling and methodological reporting across military research and practice. It is not a validated selection, injury-prediction or course-completion model and should not replace established assessments of aerobic fitness, strength, muscular endurance, load carriage or occupational performance. Prospective studies are required to determine reliability, responsiveness, incremental predictive value and criterion thresholds before the recommended framework is used to inform individual pass/fail or course-entry decisions. However, military training staff might consider including these recommended force plate tests and metrics in their pre-course physical assessment battery.

## Data Availability

The original contributions presented in the study are included in the article/Supplementary Material, further inquiries can be directed to the corresponding author/s.
