# Supplementary material for: Selecting force plate tests and metrics to complement physical preparation strategies prior to commencing strenuous military training courses
Source: Front Sports Act Living. 2026 Aug 26;8:1891717. doi: 10.3389/fspor.2026.1891717 (PMC13553802; doi:10.3389/fspor.2026.1891717)
Supplement: Supplementary file 1 [file Datasheet1.pdf]

Supplementary File: Survey Instrument

The following survey instrument was used in the study 'Optimising portable force plate practices across UK Defence rehabilitation, health and performance pathways.'

Survey Page One

The purpose of this survey is to identify how individuals with known subject matter expertise in the application of force plate technology in research and/or applied practice might use this technology as an assessment tool to inform/guide decision making criteria within military specific scenarios. Specifically, operational preparedness prior to completing a physically arduous military training exercise/course (i.e., operational fitness).

There is currently a knowledge gap within UK Defence when it comes to identifying the most important metrics to report for any given force plate test. The large quantity of metrics provided by commercial force plate providers can make identifying the most important metrics even more daunting to the inexperienced practitioner. By evaluating expert opinion, it is believed this will inform future applied and research practices across UK Defence Medical Services and the British Army.

By clicking the next button, you are confirming that you have read and understood the participant information sheet and consented to take part in this survey.

* Indicates required question

Survey Page Two

Thank you for agreeing to participate in this survey, which will help guide and develop best practice guidelines for the application of portable force plate technology within Defence settings.

Attached to the email (where you access the link to this survey) you will find a document detailing the criteria we would like you to use to inform your responses. This supporting document details a military specific performance scenario. Please use this document to guide your responses. This will ensure that all answers are standardised among survey participants.

The survey will begin when you click the ‘next’ button below.

Section 1: Your Employment and Experience Status

This section will provide survey participant demographic information relating to their job role, qualifications and experiences.

1. Which of the following most closely resembles your primary job role? * Mark only one.

Academic/Researcher - Strength and conditioning
Academic / Researcher - Physiotherapist
Applied practitioner - Strength and conditioning
Applied practitioner - Physiotherapist
Other:

2. Which country do you currently work from? * Mark only one.

Albania
Australia
Belgium
Bulgaria
Canada
Croatia
Czech Republic
Denmark
Estonia
Finland
France
Germany
Greece
Hungary
Iceland
Italy
Latvia
Lithuania
Luxenbourg
Montenegro
Netherlands
New Zealand
North Macedonia
Norway
Poland
Portugal
Romania
Slovakia
Slovenia
Spain

Sweden
Turkey
United Kingdom
United States
Other:

3. How many years of experience to you have working in academia/research? * Mark only one.

<1 year
1 to 3 years
4 to 6 years
7 to 9 years
>10 years

4. How many years of experience to you have as an applied practitioner (S&C or physiotherapy) aligned to health or performance? *Mark only one

0 years
<1 year
1 to 3 years
4 to 6 years
7 to 9 years
>10 years

5. How many years of experience do you have using force plate technology? *Mark only one.

1 to 3 years
4 to 6 years
7 to 9 years
>10 years

6. What is the highest level of academic qualification you possess? *Mark only one.

Undergraduate degree
Postgraduate degree (Masters)
PhD or Professional Doctorate
Other:

7. Which population(s) have you use force plate technology with? *Tick all that apply.

Clinical population
Elite/professional sport

General population
Recreational Athletes
Tactical populations (military, police, firefighters, paramedics etc)
Other:

8. Which force plate provider do you primarily use in your applied/research practice *Tick all that apply.

AMTI
Bertec
CONTEMPLUS
Hawkin Dynamic
Kistler
PASCO
Sparta Science
Vald (ForceDecks)

9. Have you ever worked with military personnel as a researcher or applied practitioner? *Mark only one.

Yes
No

Section 2 Force Plate Tests and Metrics to be Used During Performance Pathways
We would like to gain a better understanding of the force plate tests and metrics you would recommend to inform UK Defence rehabilitation exit criteria, health and performance pathways. We have categorised these tests as follows:

Body weight squat
Isometric test (multi-joint)
Isometric test (single-joint)
Countermovement jump (CMJ)
Squat jump
Drop vertical jump
CMJ rebound jump
Drop landings
Multi rebound jumps
Please click 'next' to continue.

Operational Performance: Physical Preparedness to Pass an Arduous Military Training Course
Elite infantry personnel are expected to comfortably pass the minimum physical employment standards (PES) outlined in the role fitness test (RFT) and soldier conditioning review (SCR). During their career, infantry personnel may request to attend

physically arduous military training exercises and courses (for example, special forces selection), often under challenging environmental conditions.

We would like you to consider the following scenario:
An infantry unit is about to commence a 4 week physically arduous training exercise in a highly mountainous region of the UK. The course involves marching long distances (>25 miles) with heavy loads (>40kg), and multiple timed best effort runs (orienteering) over shorter distances (3 to 10 miles) with reduced load (15 to 25kg). Technical and tactical activities occur during daytime and night time. Personnel are often sleep deprived and have restricted calorie intake. This course has a high failure rate due to MSK injury, voluntary withdrawal, and personnel not meeting the required standards during timed events.

UK Defence are keen to understand whether baseline force plate testing (taken within 7 days prior to the course commencing) might inform future pass/fail rates and MSKI occurrence. If force plate testing can, this would substantially reduce future training costs to the UK Ministry of Defence but also provide insightful guidance to the physical training staff, informing pre-course training material on physical preparation strategies. Everyone attending the course would have passed their RFT and SCR within 3 months of attending the arduous military training course.

We would like to know whether you think portable force plate testing could provide added value to the pre-course physical assessment criteria and if so which force plate tests and what metrics would you recommend and why?

10. Do you believe force plate derived physical assessments can be useful to inform operational readiness prior to completing an arduous training course (a pre-course selection criteria)? *Mark only one.

Yes
No, Skip to question 144

Section 3: Bodyweight Squat (Operational Performance: Pre-Course Selection Criteria)
There are many metrics derived from a bodyweight squat test. It can be overwhelming deciding which metrics are pertinent to the clinical question of interest. Your opinion regarding whether this test should be used and which metrics are considered important to inform pre-course selection criteria would be very insightful and valuable for UK Defence.

11. Do you consider a force plate derived bodyweight squat test useful to inform a pre-course selection criteria? *Mark only one.

Yes
No, Skip to question 18

Bodyweight Squat Test and Metrics
Metrics derived from open-ended body weight squat usually fall under two data categorises:
1) Force
2) Sway

Within this section you will be asked whether you recommend using this type of force plate data category (e.g., force [Yes/no]). If yes, you will then be prompted to select which metrics you recommend from a list of examples. If you select no, you will skip force-based metrics and be asked the same question but related to the next data category on the list... in this example the next question will be related to 'Sway' based metrics.

12. Would you measure FORCE related metrics from the open-ended bodyweight squat test when informing pre-course selection criteria? *Mark only one.

Yes
No

13. If Yes, which FORCE -based metric(s) would you select? *Tick all that apply.

Peak force (N)
Left limb - peak force (N)
Right limb - peak force (N)
Average force (N)
Left limb - average force (N)
Right limb - average force (N)
Left - standard deviation force (N)
Right limb - standard deviation force (N)
L/R average force (%)
L/R peak force (%)
Other

14. Would you recommend using SWAY based metrics from the open-ended bodyweight squat test when informing pre-course selection criteria? *Mark only one.

Yes
No

15. If 'Yes', which SWAY based metric(s) would you recommend? * Tick all that apply.

Left limb - sway length (cm)
Right limb - sway length (cm)
Left limb - sway range (cm)
Right limb - sway range (cm)
Left limb - sway velocity (cm.s)
Right limb - sway velocity (cm.s)
Left limb - anterior-posterior (AP) sway length (cm)
Right limb - AP sway length (cm)
Left limb - AP sway range (cm)
Right limb - AP sway range (cm)
Left limb - average AP sway velocity (cm.s)
Right limb - average AP sway velocity (cm.s)
Left limb - Medio-lateral (ML) sway length (cm)
Right limb - ML sway length (cm)
Left limb - ML sway range (cm)
Right limb - ML sway range (cm)
Left limb - average ML sway velocity (cm.s)
Right limb - average ML sway velocity (cm.s)
Other:

16. Are there any metrics NOT LISTED above that you would consider important to inform pre-course selection criteria? * Freehand response

If yes, please list below

17. From the list of metrics identified above, which FIVE metrics would you consider the most important to measure to inform pre-course selection criteria?
Please list your FIVE answers below (answers do not have to be in order of preference): * Freehand response

Section 4: Isometric Muscle Testing (Operational Performance: Pre-Course Selection Criteria)

There are many metrics derived from isometric (multi and single joint) force plate testing. It can be overwhelming deciding which metrics are pertinent to the clinical question of interest. Your opinion regarding whether this test should be used and which metrics are considered useful to inform pre-course selection criteria would be very insightful and valuable for UK Defence.

18. Do you consider force plate derived isometric muscle testing useful to inform pre-course selection criteria ?
If Yes, the next sections will ask questions related to:
1) multi joint isometric testing, and
2) single joint isometric testing

If no, you will skip these section and be asked your thoughts relating to CMJ testing. *Mark only one.

Yes
No Skip to question 41

Multi-joint Isometric Muscle T esting (Operational Performance: Pre Course Selection Criteria)
This section asks questions surrounding any recommended use of MULTI JOINT isometric muscle testing

19. Do you consider force plate derived Multi Joint isometric muscle testing useful to inform pre-course selection criteria? * Mark only one.

Yes
No Skip to question 30

Multi-joint Isometric Tests

20. Which multi joint isometric tests do you consider important to inform pre-course selection criteria? * Tick all that apply.

Isometric back squat
Isometric back squat (unilateral)
Isometric belt squat
Isometric belt squat (unilateral)
Isometric midthigh pull
Isometric midthigh pull (unilateral)
Other

Multi joint isometric test metrics
Outputs derived from multi-joint isometric tests usually fall under the following metrics:
1) Force
2) Rate of Force Development
3) Impulse
4) Time-based

Within this section you will be asked whether you recommend using the type of metric listed above (e.g., force [Yes/no]). If yes, you will asked to select which metrics you recommend using from a list of examples. If you select no, you will be asked to skip this question and instead consider the next type of output listed.

21. Would you measure FORCE related metrics from multi-joint isometric tests when informing pre-course selection criteria?

If no, select this response and skip to the next section (single joint isometric tests). *Mark only one.

Yes
No

22. If Yes, which FORCE -based metric(s) would you select? *Tick all that apply.

Absolute peak force (N)
Net peak force (N)
Relative peak force (N.kg)
Left limb - peak force (N)
Right limb - peak force (N)
Limb symmetry (L/R peak force) (%)
Force at 50 ms (N)
Force at 100 ms (N)
Force at 150 ms (N)
Force at 200 ms (N)
Force at 250 ms (N)
Force at 300 ms (N)
Left limb - force at 50 ms (N)
Left limb - force at 100 ms (N)
Left limb - force at 150 ms (N)
Left limb - force at 200 ms (N)
Left limb - force at 250 ms (N)
Left limb - force at 300 ms (N)
Right limb - force at 50 ms (N)
Right limb - force at 100 ms (N)
Right limb - force at 150 ms (N)
Right limb - force at 200 ms (N)
Right limb - force at 250 ms (N)
Right limb - force at 300 ms (N)
Other

23. Would you measure RATE OF FORCE DEVELOPMENT metrics from multi-joint isometric tests when informing pre-course selection criteria? *Mark only one.
Yes
No

24. If Yes, which RATE OF FORCE DEVELOPMENT (RFD) metric(s) would you select? *Tick all that apply.

Rate of force development 0-50 ms (N.s)
Rate of force development 0-100 ms (N.s)

Rate of force development 0-150 ms (N.s)
Rate of force development 0-200 ms (N.s)
Rate of force development 0-250 ms (N.s)
Rate of force development 0-300 ms (N.s)
Other

25. Would you measure IMPULSE metrics from multi-joint isometric tests when informing pre-course selection criteria? *Mark only one.

Yes
No

26. If yes, which IMPULSE metric(s) would you select? *Tick all that apply.

Impulse 0-100 ms (N.s)
Impulse 0-150 ms (N.s)
Impulse 0-200 ms (N.s)
Impulse 0-250 ms (N.s)
Impulse 0-300 ms (N.s)
Net impulse 0-50 ms (N.s)
Net impulse 0-100 ms (N.s)
Net impulse 0-150 ms (N.s)
Net impulse 0-200 ms (N.s)
Net impulse 0-250 ms (N.s)
Net impulse 0-300 ms (N.s)
Other

27. Would you measure TIME-BASED metrics from multi-joint isometric tests when informing pre-course selection criteria? *Mark only one.

Yes
No

28. If yes, which TIME-BASED metric(s) would you select? *Tick all that apply.

Time to peak force (s)
Other

29. From the list of multi-joint isometric test metrics identified above, which FIVE would you consider the most important to measure to inform pre-course selection criteria?
Please list your FIVE answers below (answers do not have to be in order of preference): *Freehand response

Single-Joint Isometric Tests (Operational Performance: Pre-Course Selection Criteria)

This section asks questions surrounding any recommended use of SINGLE JOINT Isometric muscle task to inform operational fitness.

Outputs derived from single-joint isometric tests usually fall under the following metrics:
1) Force
2) Rate of Force Development
3) Impulse
4) Time-based

Within this section you will be asked whether you recommend using the type of metric listed above (e.g., force [Yes/no]). If yes, you will asked to select which metrics you recommend using from a list of examples. If you select no, you will be asked to skip this question and instead consider the next type of output listed.

30. Do you consider force plate derived single Joint isometric muscle testing useful to inform pre-course selection criteria? *Mark only one.

Yes
No Skip to question 41

Single Joint isometric muscle testing to inform pre-course selection criteria?

31. Which single joint isometric tests do you consider useful to inform pre-course selection criteria? *Tick all that apply.

Isometric 90/90 hamstring test
Isometric 90/20 hamstring test
Isometric 30/30 hamstring test
Isometric 'kneeling' plantar flexion test (unilateral)
Isometric 'seated' plantar flexion test
Isometric 'seated' plantar flexion test (unilateral)
Isometric 'standing' plantar flexion test
Isometric 'standing' plantar flexion test (unilateral)
Single joint isometric test metrics
Other

32. Would you measure FORCE related metrics from single-joint isometric tests when informing pre-course selection criteria? *Mark only one.

Yes
No

33. If yes, which FORCE metric(s) would you select? *Tick all that apply.

Absolute peak force (N)
Net peak force (N)
Relative peak force (N.kg)
Left limb - peak force (N)
Right limb - peak force (N)
Limb symmetry (L/R peak force) (%)
Force at 50 ms (N)
Force at 100 ms (N)
Force at 150 ms (N)
Force at 200 ms (N)
Force at 250 ms (N)
Force at 300 ms (N)
Left limb - force at 50 ms (N)
Left limb - force at 100 ms (N)
Left limb - force at 150 ms (N)
Left limb - force at 200 ms (N)
Left limb - force at 250 ms (N)
Left limb - force at 300 ms (N)
Right limb - force at 50 ms (N)
Right limb - force at 100 ms (N)
Right limb - force at 150 ms (N)
Right limb - force at 200 ms (N)
Right limb - force at 250 ms (N)
Right - force at 300 ms (N)
Other

34. Would you measure RATE OF FORCE DEVELOPMENT metrics from single-joint isometric tests when informing pre-course selection criteria? *Mark only one.

Yes
No

35. If yes, which RATE OF FORCE DEVELOPMENT (RFD) metric(s) would you select? *Tick all that apply.

Rate of force development 0-50 ms (N.s)
Rate of force development 0-100 ms (N.s)
Rate of force development 0-150 ms (N.s)
Rate of force development 0-200 ms (N.s)
Rate of force development 0-250 ms (N.s)

Rate of force development 0-300 ms (N.s)
Other

36. Would you measure IMPULSE metrics from single-joint isometric tests when informing pre-course selection criteria? *Mark only one.

Yes
No

37. If yes, which IMPULSE metric(s) would you select? *Tick all that apply.

Impulse 0-100 ms (N.s)
Impulse 0-150 ms (N.s)
Impulse 0-200 ms (N.s)
Impulse 0-250 ms (N.s)
Impulse 0-300 ms (N.s)
Net impulse 0-50 ms (N.s)
Net impulse 0-100 ms (N.s)
Net impulse 0-150 ms (N.s)
Net impulse 0-200 ms (N.s)
Net impulse 0-250 ms (N.s)
Net impulse 0-300 ms (N.s)
Other

38. Would you measure TIME-BASED metrics from single-joint isometric tests when informing pre-course selection criteria? *Mark only one.

Yes
No

39. If yes, which TIME-BASED metric(s) would you select? *Tick all that apply.

Time to peak force (s)
Other

40. From the list of metrics identified above, which FIVE would you consider the most important to measure to inform pre-course selection criteria? Please list your FIVE answers below (answers do not have to be in order of preference): *Freehand response

Countermovement Jump T esting (Operational Performance: Pre-Course Selection Criteria)

There are many metrics derived from the countermovement jump. It can be overwhelming to decide which metrics are pertinent to the clinical question of interest.

Your opinion regarding whether this test should be used and which metrics are considered useful to inform pre-course selection criteria would be very insightful and valuable for UK Defence.

41. Do you consider the countermovement jump a useful test to inform pre-course selection criteria? *Mark only one.

Yes
No Skip to question 59

Section 5: Countermovement Jump Test and Metrics
Outputs derived from CMJ tests usually fall under the following metrics:
1) Outcome
2) Force
3) Power
4) Velocity
5) Rate of Force Development
6) Impulse
7) Time-based

Within this section you will be asked whether you recommend using the type of metric listed above (e.g., force [Yes/no]). If yes, you will asked to select which metrics you recommend using from a list of examples. If you select no, you will be asked to skip this question and instead consider the next type of output listed... in this example the next question will be related to 'rate of force development' based metrics.

42. Would you measure OUTCOME metrics from the countermovement jump test when informing pre-course selection criteria? *Mark only one.

Yes
No

43. If yes, which OUTCOME metric(s) would you select? *Tick all that apply.

Jump height (cm)
Jump momentum (kg*m.s)
Take off velocity (m.s)
Reactive strength index (modified)
Flight time (s)
Other

44. Would you measure FORCE metrics from the countermovement jump test when informing pre-course selection criteria? * Mark only one.

Yes
No

45. If yes, which FORCE metric(s) would you select? *Tick all that apply.

Peak landing force (N)
Average landing force (N)
Average braking/eccentric force (N)
Peak braking/eccentric force (N)
Average relative braking/eccentric force (N.kg)
Peak relative braking/eccentric force (N.kg)
Left limb - average braking/eccentric force (N)
Right limb - average braking/eccentric force (N)
Left limb - peak braking/eccentric force (N)
Right limb - peak braking/eccentric force (N)
L/R average braking/eccentric force (%)
L/R peak braking/eccentric force (%)
Peak relative landing force (N.kg)
Left limb - average landing force (N)
Right limb - average landing force (N)
Left limb - force at peak landing force (N)
Right limb - force at peak landing force (N)
L/R average landing force (%)
L/R peak landing force (%)
Left limb - average propulsive/concentric force (N)
Right limb - average propulsive/concentric force (N)
Left limb - force at peak propulsive/concentric force (N)
Right limb - force at peak propulsive/concentric force (N)
L/R average propulsive/concentric force (%)
L/R peak propulsive/concentric force (%)
Average propulsion/concentric force (N)
Peak propulsive/concentric force (N)
Peak net propulsive/concentric force (N)
Average relative propulsive/concentric force (N.kg)
Peak relative propulsive/concentric force (N.kg)
Force at min displacement (N)
Relative force at min displacement (N.kg)
Other

46. Would you measure POWER metrics from the countermovement jump test when informing pre-course selection criteria? *Mark only one.

Yes
No

47. If yes, which POWER metric(s) would you select? *Tick all that apply.

Peak propulsive/concentric power (W)
Net peak propulsive/concentric power (W)
Peak relative propulsive/concentric power (W.kg)
Average propulsive/concentric power (W)
Average relative propulsive/concentric power (W)
Peak braking/eccentric power (W)
Peak relative braking/eccentric power (W.kg)
Average braking/eccentric power (W)
Average relative breaking/eccentric power (W.kg)
Other

48. Would you measure VELOCITY metrics from the countermovement jump test when informing pre-course selection criteria? *Mark only one.

Yes
No

49. If yes, which VELOCITY metric(s) would you select? *Tick all that apply.

Average propulsive/concentric velocity (m.s)
Average braking/eccentric velocity (m.s)
Peak braking/eccentric velocity (m.s)
Other

50. Would you measure RATE OF FORCE DEVELOPMENT metrics from the countermovement jump test when informing pre-course selection criteria? *Mark only one.

Yes
No

51. If yes, which RATE OF FORCE DEVELOPMENT (RFD) metric(s) would you select? *Tick all that apply.

Left limb - average braking/eccentric RFD
Right limb - average braking/eccentric RFD
L/R average breaking/eccentric RFD (%)

Braking/eccentric RFD (N.s)
Other

52. Would you measure IMPULSE metrics from the countermovement jump test when informing pre-course selection criteria? * Mark only one.

Yes
No

53. If yes, which IMPULSE metric(s) would you select? *Tick all that apply.

Impulse ratio
L/R braking/eccentric impuse index (%)
Relative braking/eccentric impulse (N.s)
Relative braking/eccentric net impulse (N.s.kg)
Braking/eccentric impulse (N.s)
Braking/eccentric net impulse (N.s)
Positive impulse (N.s)
Positive net impulse (N.s)
L/R landing impulse index (%)
L/R propulsive/concentric impulse index (%)
Propulsive/concentric impulse (N.s)
Propulsive/concentric net impulse (N.s)
Relative propulsive/concentric impulse (N.s.kg)
Relative propulsive/concentric net impulse (N.s.kg)
Landing impulse (N.s)
Other

54. Would you measure TIME-BASED metrics from the countermovement jump test when informing pre-course selection criteria? *Mark only one.

Yes
No

55. If yes, which TIME-BASED metric(s) would you select? *Tick all that apply.

Time to takeoff / contraction time / movement time(s)
Unweighted phase duration (s)
Unweighted phase (%)
Braking/eccentric phase duration (s)
Braking/eccentric phase (%)
Propulsive/concentric phase duration (s)
Propulsive/concentric phase (%)
Time to stabilisation (s)

Time to peak force (s)
Propulsive/concentric force duration (s)
Time to peak power (s)
Landing phase duration (s)
Other

56. Would you measure OTHER metrics from the countermovement jump test when informing pre-course selection criteria? *Mark only one.

Yes
No

57. If yes, which OTHER metric(s) derived would you select? *Tick all that apply.

Countermovement depth (m)
Force-velocity AUC (N.m.s.kg))
Landing performance index
Other

58.From the list of metrics identified above, which FIVE would you consider the most important to measure to inform pre-course selection criteria? Please list your FIVE answers below (answers do not have to be in order of preference): *Freehand response

Section 6: Squat Jump Testing (Operational Performance: Pre-Course Selection Criteria)

There are many metrics derived from the squat jump test. It can be overwhelming deciding which metrics are pertinent to the clinical question of interest. Your opinion regarding whether this test should be used and which metrics are considered useful to inform pre-course selection criteria would be very insightful and valuable for UK Defence.

59. Do you consider squat jump a useful test to inform pre-course selection criteria? * Mark only one.

Yes
No Skip to question 75

Squat Jump Test and Metrics

Outputs derived from Squat Jump test usually fall under the following metrics:
1) Outcome
2) Force
3) Power
4) Velocity

5) Impulse
6) Time-based
Within this section you will be asked whether you recommend using the type of metric listed above (e.g., force [Yes/no]). If yes, you will asked to select which metrics you recommend using from a list of examples. If you select no, you will be asked to skip this question and instead consider the next type of output listed.

60. Would you measure OUTCOME metrics from the squat jump test when informing pre-course selection criteria? *Mark only one.

Yes
No

61. If yes, which OUTCOME metric(s) would you select? *Tick all that apply.

Jump height (cm)
Jump momentum (kg*m.s)
Take off velocity (m.s)
Time to takeoff / contraction time / movement time (s)
Flight time (s)
Other

62. Would you measure FORCE metrics from the squat jump test when informing pre-course selection criteria? *Mark only one.

Yes
No

63. If yes, which FORCE metric(s) would you select? *Tick all that apply.

Left limb - average landing force (N)
Right - average landing force (N)
Left limb - force at peak landing force (N)
Right - force at peak landing force (N)
L/R average landing force (%)
L/R peak landing force (%)
Average landing force (N.m)
Peak landing force (N)
Peak relative landing force (N.kg)
Left limb - average propulsive/concentric force (N)
Right limb - average propulsive/concentric force (N)
L/R average propulsive/concentric force (%)
Left limb - force at peak propulsive/concentric force (N)
Right limb - force at peak propulsive/concentric force (N)

L/R peak propulsive/concentric force (%)
Average propulsion/concentric force (N)
Average relative propulsive/concentric force (N.kg)
Peak propulsive/concentric force (N)
Peak net propulsive/concentric force (N)
Peak relative propulsive/concentric force (N.kg)
Other

64. Would you measure POWER metrics from the squat jump test when informing pre-course selection criteria? *Mark only one.

Yes
No

65. If yes, which POWER metric(s) would you select? *Tick all that apply.

Peak propulsive/concentric power (W)
Net peak propulsive/concentric power (W)
Peak relative propulsive/concentric power (W.kg)
Average propulsive/concentric power (W)
Average relative propulsive/concentric power (W.kg)
Other

66. Would you measure VELOCITY metrics from the squat jump test when informing pre-course selection criteria? *Mark only one.

Yes
No

67. If yes, which VELOCITY metric(s) derived would you select? *Tick all that apply.

Average propulsive/concentric velocty (m.s)
Other

68. Would you measure IMPULSE metrics from the squat jump test when informing pre-course selection criteria? *Mark only one.

Yes
No

69. If yes, which IMPULSE metric(s) derived would you select? *Tick all that apply.

L/R landing impulse index (%)
L/R propulsive/concentric impulse index (%)

Relative propulsive/concentric impulse (N.s.kg)
Relative propulsive/concentric net impulse (N.s.kg)
Propulsive/concentric impulse (N.s)
Propulsive/concentric net impulse (N.s)
Landing impulse (N.s)
Other

70. Would you measure TIME-BASED metrics from the squat jump test when informing pre-course selection criteria? *Mark only one.

Yes
No

71. If yes, which TIME-BASED metric(s) would you select? *Tick all that apply.

Time to stabilisation (s)
Time to peak force (s)
Time to peak power (s)
Propulsive phase duration (s)
Landing phase duration (s)
Other

72. Would you measure OTHER metrics from the squat jump test when informing pre-course selection criteria? *Mark only one.

Yes
No

73. If yes, which OTHER metric(s) derived would you select? *Tick all that apply.

Landing performance index
Other

74. From the list of metrics identified above, which FIVE would you consider the most important metrics to measure to inform pre-course selection criteria?
Please list your FIVE answers below (answers do not have to be in order of preference): *Freehand response

Section 7: Drop Jump T esting (Operational Performance: Pre-Course Selection Criteria)

There are many metrics derived from a drop jump test. It can be overwhelming deciding which metrics are pertinent to the clinical question of interest. Your opinion regarding

whether this test should be used and which metrics are considered useful to inform pre-course selection criteria would be very insightful and valuable for UK Defence.

75. Do you consider the drop jump test an useful test to inform pre-course selection criteria? *Mark only one.

Yes
No Skip to question 94

Drop Jump T est and Metrics
Outputs derived from drop jump test usually fall under the following metrics:
1) Outcome
2) Force
3) Power
4) Velocity
5) Rate of Force Development
6) Impulse
7) Time-based

Within this section you will be asked whether you recommend using the type of metric listed above (e.g., force [Yes/no]). If yes, you will asked to select which metrics you recommend using from a list of examples. If you select no, you will be asked to skip this question and instead consider the next type of output listed.

76. Would you measure OUTCOME metrics from the drop jump test when informing pre-course selection criteria? *Mark only one.

Yes
No

77. If yes, which OUTCOME metric(s) would you select? *Tick all that apply.

Reactive strength index
Jump momentum (kg*m.s)
Jump height (cm)
Flight time (s)
Takeoff velocity (m.s)
Drop height (cm)
Other

78. Would you measure FORCE metrics from the drop jump test when informing pre-course selection criteria? *Mark only one.

Yes
No

79. If yes, which FORCE metric(s) would you select? *Tick all that apply.

Left limb - average braking/eccentric force (N)
Right limb - average braking/eccentric force (N)
Left limb - force at peak braking/eccentric force (N)
Right limb - force at peak braking/eccentric force (N)
L/R average braking/eccentric force (%)
L/R peak braking/eccentric force (%)
Average braking/eccentric force (N)
Peak braking/eccentric force (N)
Average relative braking/eccentric force (N.kg)
Peak relative braking/eccentric force (N.kg)
Left limb - average landing force (N)
Right limb - average landing force (N)
Left limb - force at peak landing force (N)
Right limb - force at peak landing force (N)
L/R average landing force (%)
L/R peak landing force (%)
Average landing force (N)
Peak landing force (N)
Relative peak landing force (N)
Left limb - average propulsive/concentric force (N)
Right limb - average propulsive/concentric force (N)
Left limb - force at peak propulsive/concentric force (N)
Right limb - force at peak propulsive/concentric force (N)
L/R average propulsive/concentric force (%)
L/R peak propulsive/concentric force (%)
Average propulsive/concentric force (N)
Peak propulsive/concentric force (N)
Force at min displacement (N)
Relative force at min displacement (N.kg)
Other

80. Would you measure POWER metrics from the drop jump test when informing pre-course selection criteria? *Mark only one.

Yes
No

81. If yes, which POWER metric(s) would you select? * Tick all that apply.

Average relative braking/eccentric power (W.kg)
Peak relative braking/eccentric power (W.kg)
Average braking/eccentric power (W)
Peak braking/eccentric power (W)
Average relative propulsive/concentric power (W.kg)
Peak relative propulsive/concentric power (W.kg
Average propulsive/concentric power (W)
Peak propulsive/concentric power (W)
Other

82. Would you measure VELOCITY metrics from the drop jump test when informing pre-course selection criteria? *Mark only one.

Yes
No

83. If yes, which VELOCITY metric(s) would you select? *Tick all that apply.

Average propulsive/concentric velocity (m.s)
Average braking/eccentric velocity (m.s)
Other

84. Would you measure RATE OF FORCE DEVELOPMENT (RFD) metrics from the drop jump test when informing pre-course selection criteria? *Mark only one.

Yes
No

85. If yes, which RATE OF FORCE DEVELOPMENT metric(s) would you select? *Tick all that apply.

Left limb - average braking/eccentric RFD (N.s)
Right limb - average braking/eccentric RFD (N.s)
L/R average braking/eccentric RFD (%)
Braking/eccentric RFD (N.s)
Other

86. Would you measure IMPULSE metrics from the drop jump test when informing pre-course selection criteria? *Mark only one.
Yes
No

87. If yes, which IMPULSE metric(s) would you select? *Tick all that apply.

L/R braking/eccentric impulse index (%)
Relative braking/eccentric impulse (N.s.kg)
Relative braking/eccentric net impulse (N.s.kg)
Braking/eccentric impulse (N.s)
Braking/eccentric net impulse (N.s)
Positive impulse (N.s)
Positive net impulse (N.s)
L/R landing impulse index (%)
relative propulsive/concentric impulse (N.s.kg)
Relative propulsive/concentric net impulse (N.s.kg)
Propulsive/concentric impulse (N.s)
Propulsive/concentric net impulse (N.s)
Impulse ratio
Other

88. Would you measure TIME-BASED metrics from the drop jump test when informing pre-course selection criteria? *Mark only one.

Yes
No

89. If yes, which TIME-BASED metric(s) would you select? *Tick all that apply.

Average braking/eccentric force (N)
Time to peak braking/eccentric force (s)
Braking/eccentric phase duration (s)
Braking/eccentric phase %
Propulsive/concentric phase duration (s)
Propulsive/concentric phase %
Landing phase duration (s)
Time to stabilisation (s)
Other

90. Would you measure OTHER metrics from the drop jump test when informing pre-course selection criteria? *Mark only one.

Yes
No

91. If yes, which OTHER metric(s) would you select? *Tick all that apply.

Peak impact

Spring like correlation
Landing stiffness (N.m)
Stiffness during rebound phase (N.m)
Countermovement depth (cm)
Landing performance index
Other

92. From the list of metrics identified above, which FIVE would you consider the most important metrics to measure to inform the pre-course selection criteria?
Please list your FIVE answers below (answers do not have to be in order of preference): *Freehand response

93. What box height do you feel is important to use when conducting a drop vertical jump test to inform pre-course selection criteria? *Tick all that apply.

Bilateral DVJ - 20cm
Bilateral DVJ - 30cm
Bilateral DVJ - 40cm
Bilateral DVJ - 50cm
Bilateral DVJ - 60cm
Unilateral DVJ - 20cm
Unilateral DVJ - 30cm
Unilateral DVJ - 40cm
Unilateral DVJ - 50cm
Unilateral - DVJ - 60cm
Other

Section 8: Countermovement jump (CMJ) Rebound Jump Testing (Operational Performance: Pre-Course Selection Criteria
There are many metrics derived from a CMJ Rebound jump test. It can be overwhelming deciding which metrics are pertinent to the clinical question of interest. Your opinion regarding whether this test should be used and which metrics are considered useful to inform pre-course selection criteria would be very insightful and valuable for UK Defence.

94. Do you believe CMJ rebound jump tests are useful to inform pre-course selection criteria? * Mark only one.

Yes
No Skip to question 110

CMJ Rebound Jump Tests and Metrics
Outputs derived from CMJ Rebound Jump test usually fall under the following metrics:

1) Outcome
2) Force
3) Power
4) Rate of Force Development
5) Impulse
6) Time-based
Within this section you will be asked whether you recommend using the type of metric listed above (e.g., force [Yes/no]). If yes, you will asked to select which metrics you recommend using from a list of examples. If you select no, you will be asked to skip this question and instead consider the next type of output listed.

95. Would you measure OUTCOME metrics from the CMJ Rebound Jump test when informing pre-course selection criteria? *Mark only one.

Yes
No

96. If yes, which OUTCOME metric(s) would you select? *Tick all that apply.

CMJ - modified reactice strength index (mRSI)
Rebound - Reactive strength index (RSI)
CMJ - jump momentum (kg*m.s)
Rebound - jump momentum (kg*m.s)
CMJ - jump height (cm)
Rebound - jump height (cm)
Rebound - flight time (s)
Other

97. Would you measure FORCE metrics from the CMJ Rebound Jump test when informing pre-course selection criteria? *Mark only one.

Yes
No

98. If yes, which FORCE metric(s) would you select? *Tick all that apply.

CMJ - Left limb - average braking/eccentric force (N)
CMJ - Right limb - average braking/eccentric force (N)
CMJ - L/R average braking/eccentric force (%)
CMJ - average braking/eccentric force (N)
CMJ - peak braking/eccentric force (N)
CMJ - average relative braking/eccentric force (N.kg)
CMJ - peak relative braking/eccentric force (N.kg)
Rebound - Left limb - average braking/eccentric force (N)

Rebound - Right limb - average braking/eccentric force (N)
Rebound - L/R average braking/eccentric force (%)
Rebound - average braking/eccentric force (N)
Rebound - peak braking/eccentric force (N)
Rebound - average relative braking/eccentric force (N.kg)
Rebound - peak relative braking/eccentric force (N.kg)
Rebound - time to peak braking/eccentric force (s)
Average landing force (N)
Peak landing force (N)
Relative peak landing force (N.kg)
Left limb - average landing force (N)
Right limb - average landing force (N)
L/R average landing force (%)
L/R peak landing force (%)
CMJ - Left limb - average propulsive/concentric force (N)
CMJ – Right limb - average propulsive/concentric force (N)
CMJ - L/R average propulsive/concentric force (%)
CMJ - average propulsive/concentric force (N)
CMJ - peak propulsive/concentric force (N)
CMJ - average relative propulsive/concentric force (N.kg)
CMJ - peak relative propulsive/concentric force (N.kg)
Rebound - L/R average propulsive/concentric force (%)
Rebound - Left limb - average propulsive/concentric force (N)
Rebound - Right limb - average propulsive/concentric force (N)
Rebound - average propulsive/concentric force (N)
Rebound - peak propulsive/concentric force (N)
Rebound - average relative propulsive/concentric force (N.kg)
Rebound - peak relative propulsive/concentric force (N.kg)
CMJ - force at min displacement (N)
CMJ - relative force at min displacement (N)
Rebound - force at min displacement (N)
Rebound - relative force at min displacement (N)
Other

99. Would you measure POWER metrics from the CMJ Rebound Jump test when informing pre-course selection criteria? *Mark only one.

Yes
No

100. If yes, which POWER metric(s) would you select? *Tick all that apply.

CMJ - average relative braking/eccentric power (W.kg)
CMJ - peak relative braking/eccentric power (W.kg)

CMJ - average braking/eccentric power (W)
CMJ - peak braking/eccentric power (W)
Rebound - average relative braking/eccentric power (W.kg)
Rebound - peak relative braking/eccentric power (W.kg)
Rebound - average braking/eccentric power (W)
Rebound - peak braking/eccentric power (W)
CMJ - average relative propulsive/concentric power (W.kg)
CMJ - peak relative propulsive/concentric power (W.kg)
CMJ - average propulsive/concentric power (W)
CMJ - peak propulsive/concentric power (W)
Rebound - average relative propulsive/concentric power (W.kg)
Rebound - peak relative propulsive/concentric power (W.kg)
Rebound - average propulsive/concentric power (W)
Rebound - peak propulsive/concentric power (W)
Other:

101. Would you measure RATE OF FORCE DEVELOPMENT (RFD) metrics from the CMJ Rebound Jump test when informing pre-course selection criteria? *Mark only one.

Yes
No

102. If yes, which RATE OF FORCE DEVELOPMENT (RFD) metric(s) would you select? * Tick all that apply

CMJ - braking/eccentric RFD (N.s)
Other.

103. Would you measure IMPULSE metrics from the CMJ Rebound Jump test when informing pre-course selection criteria? * Mark only one.

Yes
No

104. If yes, which IMPULSE metric(s) would you select? * Tick all that apply.
CMJ - L/R braking/eccentric impulse index (%)
CMJ - relative braking/eccentric impulse (N.s.kg)
CMJ - relative braking/eccentric net impulse (N.s.kg)
Rebound - L/R braking/eccentric impulse index (%)
Rebound - relative braking/eccentric impulse (N.s.kg)
Rebound - relative braking/eccentric net impulse (N.s.kg)
Rebound - braking/eccentric impulse (N.s)

Rebound - braking/eccentric net impulse (N.s)
CMJ - positive impulse (N.s)
CMJ - positive net impulse (N.s)
Rebound - positive impulse (N.s)
Rebound - positive net impulse (N.s)
L/R Landing impulse index (%)
CMJ - L/R propulsive/concentric impulse index (%)
CMJ - relative propulsive/concentric impulse (N.s.kg)
CMJ - relative propulsive/concentric net impulse (N.s.kg)
CMJ - propulsive/concentric impulse (N.s)
CMJ - propulsive/concentric net impulse (N.s)
Rebound - L/R propulsive/concentric impulse index (%)
Rebound - relative propulsive/concentric impulse (N.s.kg)
Rebound - relative propulsive/concentric net impulse (N.s.kg)
Rebound - propulsive/concentric impulse (N.s)
Rebound - propulsive/concentric net impulse (N.s)
CMJ - impulse ratio
Rebound - impulse ratio
Other

105. Would you measure TIME-BASED metrics from the CMJ Rebound Jump test when informing pre-course selection criteria? *Mark only one.

Yes
No

106. If yes, which TIME-BASED metric(s) would you select? *Tick all that apply.

CMJ - time to takeoff (s)
Rebound - time to takeoff (s)
Rebound - ground contact time (s)
Rebound - flight time (s)
Time to stabilisation (s)
Landing phase duration (s)
Other

107. Would you measure OTHER metrics from the CMJ Rebound Jump test when informing pre-course selection criteria? *Mark only one.

Yes
No

108. If yes, which OTHER metric(s) would you select? * Tick all that apply.

Rebound - spring like correlation
Landing stiffness (N.m)
Countermovement depth (cm)
Rebound - stiffness (N.m)
Rebound - countermovement depth (cm)
Landing performance index
Other.

109. From the list of metrics identified above, which TEN would you consider the most important metrics to measure to inform the pre-course selection criteria?
Please list your TEN answers below (answers do not have to be in order of preference) *Freehand response

Section 9: Drop Landing T asks (Operational Performance: Pre-Course Selection Criteria)
There are many metrics derived from a Drop Landing tests. It can be overwhelming deciding which metrics are pertinent to the clinical question of interest. Your opinion regarding whether this test should be used and which metrics are considered useful to inform pre-course selection criteria would be very insightful and valuable for UK Defence.

110. Do you consider the drop landing tasks to be a useful test to inform pre-course selection criteria? *Mark only one.

Yes
No Skip to question 127

Drop Landing T asks and Metrics
Outputs derived from drop landing task usually fall under the following metrics:
1) Outcome
2) Force
3) Power
4) Velocity
5) Rate of Force Development
6) Time-based

Within this section you will be asked whether you recommend using the type of metric listed above (e.g., force [Yes/no]). If yes, you will asked to select which metrics you recommend using from a list of examples. If you select no, you will be asked to skip this question and instead consider the next type of output listed.

111. Would you measure OUTCOME metrics from a drop landing task when informing pre-course selection criteria? *Mark only one.

Yes
No

112. If yes, which OUTCOME metric(s) would you select? * Tick all that apply.

Drop height (cm)
Stability depth (cm)
Other.

113. Would you measure FORCE metrics from the Drop Landing task when informing pre-course selection criteria? *Mark only one.

Yes
No

114. If yes, which FORCE metric(s) would you select? *Tick all that apply.

Left limb - average impact force (N)
Right limb - average impact force (N)
Left limb - force at peak force (N)
Right limb - force at peak force (N)
L/R peak force (%)
L/R average impact force (%)
Average impact force (N)
Peak force (N)
peak relative force (N.kg)
Left limb - average stabilisation force (N)
Right limb - average stabilisation force (N)
L/R average stabilisation force (%)
Average stabilisation force (N)
Other

115. Would you measure POWER metrics from the Drop Landing task when informing pre-course selection criteria? *Mark only one.

Yes
No

116. If yes, which POWER metric(s) would you select? * Tick all that apply.

Average relative impact power (W.kg)
Peak relative impact power (W.kg)
Average impact power (W)

Peak impact power (W)
Average relative stabilisation power (W.kg)
Peak relative stabilisation power (W.kg)
Average stabilisation power (W)
Peak stabilisation power (W)
Other

117. Would you measure VELOCITY metrics from the Drop Landing task when informing pre-course selection criteria? *Mark only one.

Yes
No

118. If yes, which VELOCITY metric(s) would you select? * Tick all that apply.

Average impact velocity (m.s)
Average stabilisation velocity (m.s)
Other.

119. Would you measure RATE OF FORCE DEVELOPMENT (RFD) metrics from the Drop Landing task when informing pre-course selection criteria? *Mark only one

Yes
No

120. If yes, which RATE OF FORCE DEVELOPMENT (RFD) metric(s) would you select? *Tick all that apply.

Left limb - average impact RFD (N.s)
Right limb - average impact RFD (N.s)
Impact RFD (N.s)
Other.

121. Would you measure TIME-BASED metrics from the Drop Landing task when informing pre-course selection criteria? * Mark only one.

Yes
No

122. If yes, which TIME-BASED metric(s) would you select? * Tick all that apply.

Impact phase duration (s)
Impact phase %
Stabilisation phase duration (s)

Time to stabilisation (s)
Landing phase duration (s)
Other

123. Would you measure OTHER metrics from the Drop Landing task when informing pre-course selection criteria? *Mark only one.

Yes
No

124. If yes, which OTHER metric(s) would you select? * Tick all that apply.

Stiffness (N.m)
Landing performance index
Landing impulse (N.s)
Other

125. From the list of metrics identified above, which FIVE would you consider the most important metrics to measure to inform the pre-course selection criteria?
Please list your FIVE answers below (answers do not have to be in order of preference): * Freehand response

126. What box height do you feel is important to use when conducting a drop landing task to inform pre-course selection criteria? *Tick all that apply.

Bilateral landing - 20cm
Bilateral landing - 30cm
Bilateral landing - 40cm
Bilateral landing - 50cm
Bilateral landing - 60cm
Bilateral landing - 70cm
Bilateral landing - 80cm
Bilateral landing - 90cm
Bilateral landing - 100c m
Unilateral landing - 20cm
Unilateral landing - 30cm
Unilateral landing - 40cm
Unilateral landing - 50cm
Unilateral - landing - 60cm
Other

Section 10: Multi Rebound Jump/Hop Tests (Operational Performance: Pre-Course Selection Criteria)

There are many tests categorised as a multi rebound jump/hop test and many metrics derived from these tests. It can be overwhelming deciding which metrics are pertinent to the clinical question of interest. Your opinion regarding whether this test should be used and which metrics are considered useful to inform pre-course selection criteria would be very insightful and valuable for UK Defence.

127. Do you consider multi rebound jump tests to be useful test to inform pre-course selection criteria? *Mark only one.

Yes
No Skip to question 137

Multi Rebound Jump/Hop Tests and Metrics
Outputs derived from Multi Rebound Jump/Hop test usually fall under the following metrics:
1) Outcome
2) Force
3) Time-based
Within this section you will be asked whether you recommend using the type of metric listed above (e.g., force [Yes/no]). If yes, you will asked to select which metrics you recommend using from a list of examples. If you select no, you will be asked to skip this question and instead consider the next type of output listed.

128. Which multi rebound jump/hop tests is most useful to use to inform pre-course selection criteria? *Tick all that apply.

10/5 rebound
5/3 rebound
Scandinavian rebound
Pogo jumps / hops
Bosco jump
Other

129. Would you measure OUTCOME metrics from a multi-rebound jump/hop test when informing pre-course selection criteria? *Mark only one.

Yes
No

130. If yes, which OUTCOME metric(s) would you select? *Tick all that apply.

Peak jump reactive strength index (RSI)
Peak RSI
Average RSI

Top 3 jumps/hops average RSI
Top 3 jumps/hops peak RSI
Top 5 jumps/hops average RSI
Top 5 jumps/hops peak RSI
Peak jump height (cm)
Average jump height (cm)
Top 3 jumps/hops average jump height (cm)
Top 5 jumps/hops average jump height (cm)
Other

131. Would you measure FORCE metrics from a multi-rebound jump/hop test when informing pre-course selection criteria? *Mark only one.

Yes
No

132. If yes, which FORCE metric(s) would you select? *Tick all that apply

Average force (N)
Peak force (N)
L/R average force (%)
L/R peak force (%)
Other.

133. Would you measure TIME-BASED metrics from a multi-rebound jump/hop test when informing pre-course selection criteria? *Mark only one.

Yes
No

134. If yes, which TIME-BASED metric(s) would you select? * Tick all that apply.

Total contact time (s)
Total flight time (s)
Top 3 jumps/hops average contact time (s)
Top 5 jumps/hops average contact time (s)
Average contact time (s)
Other.

135. From the list of metrics identified above, which five would you consider the most important metrics to record to inform the pre-course selection criteria?
Please list your FIVE answers below (answers do not have to be in order of preference): *Freehand response

136.Are there any other force plate tests not listed that you would consider to be a useful force plate test to inform pre-course selection criteria?
If yes, please provide the NAME of the test and your top FIVE recommended metrics derived from this test. *Freehand response

Section 11: Practical Considerations (Operational Performance: Pre-Course Selection Criteria)

137. If portable force plates were to be integrated into UK Defence practice, there will likely be time constraints associated with their administration (for example, strict management of large numbers during annual fitness testing).
If faced with a scenario where the practitioner is only provided with enough time to use THREE force plate tests to inform pre-course selection criteria, which THREE tests would you recommend? *Tick all that apply.

Bodyweight squat
Isometric back squat
Isometric belt squat
Isometric midthigh pull
Isometric 90/90 hamstring test
Isometric 90/20 hamstring test
Isometric 30/30 hamstring test
Isometric 'kneeling' plantar flexion test
Isometric 'seated' plantar flexion test
Isometric 'standing' plantar flexion test
Countermovement jump
Squat jump
Drop jump
CMJ rebound jump
Drop landing task
10 / 5 rebound
5 / 3 rebound
Scandinavian rebound
Pogo jumps / hops
Bosco jump
Other.

138. Can you please provide a short rationale for why you selected the THREE force plate tests above to inform pre-course selection criteria? *Freehand response

'LOADED' Force Plate T ests (Operational Performance: Pre-Course Selection Criteria)

Military personnel are often required to wear military kit during operational exercises. For example, helmet, rifle, webbing, day sack, military boots (combined weight typically 20-25kg).

139. Would you consider loaded force plate testing useful to inform pre-course selection criteria? *Mark only one.

Yes
No

140. Would you consider any of the following tests useful to perform under loaded conditions to inform pre-course selection criteria? *Tick all that apply.

CMJ
Squat jump
Other

141. Would any of the loaded tests featured above replace any of the top three recommended force plate tests you listed at the end of the previous section?

If yes, please name the loaded test you recommend and which test it would replace from the previous section?
If no, leave blank.

Ratio Based Metrics to Inform Performance Pathways
142. Would any of the following ratio-based metrics be considered useful to collect/analyse/report during pre-course selection? *Mark only one.

Dynamic strength index/deficit
Eccentric utilisation ratio
Other

143. If you said yes to the use of the Dynamic Strength Index/Deficit, what tests and metrics would you use in your calculations? *Freehand response

Section 12: Operational Performance: Pre-Course Selection - Final Comments
This concludes all questions related to the integration of force plate technology into pre-course selection.

144. If you would like to provide additional thoughts/comments relating to this section, please detail them below. *Freehand response

If not, please skip to the next section.

Please check your responses and submit.

END OF SURVEY

This content is neither created nor endorsed by Google.
