# Supplementary material for: Selecting force plate tests and metrics to complement physical preparation strategies prior to commencing strenuous military training courses
Source: Front Sports Act Living. 2026 Aug 26;8:1891717. doi: 10.3389/fspor.2026.1891717 (PMC13553802; doi:10.3389/fspor.2026.1891717)
Supplement: Supplementary file 2 [file Datasheet2.pdf]

Supplementary File 1. Anonymised Responses to Survey Questions

Table 1. Body weight

Questions	Responder 1	2	3	4	5	6	7	8	9	10
Do you believe force plate derived physical assessments can be useful to inform operational readiness prior to completing an arduous training course (a pre-course selection criteria)?	Yes	Yes	Yes	Yes	Yes	Yes	Yes	Yes	Yes	Yes
Do you consider a force plate derived **bodyweight squat test** appropriate / useful to inform a pre-course selection criteria?	Yes	No	No	No	No	No	Yes	Yes	No	No
Would you measure **FORCE related metrics** from the **open-ended bodyweight squat test** when informing **pre-course selection criteria**?	Yes						Yes	Yes		
If Yes, which **FORCE-based metric(s)** would you select?	Peak force (N), L/R peak force (%)						Non-injured limb - standard deviation force (N), L/R average force (%), L/R peak force (%)	Peak force (N), Injured limb - peak force (N), Non-injured limb - peak force (N), Average force (N), L/R average force (%), L/R peak force (%)		
If 'Yes', which **SWAY based metric(s)** would you recommend?							Injured limb - sway length (cm), Injured limb - AP sway range (cm), Injured limb - ML sway range (cm)			

Are there any metrics **NOT LISTED** above that you would consider appropriate / useful to inform **pre-course selection criteria**?<br>*If yes, please list below*	none						N/A			
From the list of metrics identified above, which **FIVE metrics** would you consider the **most essential** to measure to inform **pre-course selection criteria**?<br><br>**Please list your FIVE answers below** (answers do not have to be in order of preference):	Peak force (N), L/R peak force (%), Average force (N)						1. L/R peak force (%): 2. L/R average force (%); 3. Injured limb medio-lateral sway range; 4. Injured limb - sway length; 5. Injured limb anterior-posterior sway range	Peak force, injured limb peak force, non-injured limb peak force, LR peak force asymmetry, average force		

Table S2 Multijoint Isometrics Tests										
	Responder									
Questions	1	2	3	4	5	6	7	8	9	10
Do you consider force plate derived **Multi Joint isometric** muscle testing appropriate / useful to inform **pre-course selection criteria**?	Yes	Yes	Yes	Yes	No	Yes	Yes	Yes	Yes	Yes
Which **multi joint isometric tests** do you consider appropriate / useful to inform **pre-course selection criteria**?	Isometric belt squat, Isometric midthigh pull, Isometric midthigh pull (unilateral)	Isometric back squat, Isometric midthigh pull	Isometric belt squat	Isometric belt squat, Isometric midthigh pull		Isometric back squat	Isometric belt squat, Isometric midthigh pull	Isometric belt squat, Isometric midthigh pull	Isometric back squat, Isometric midthigh pull	Isometric back squat, Isometric midthigh pull
Would you measure **FORCE related metrics** from multi-joint isometric tests when informing **pre-course selection criteria**?	Yes	Yes	Yes	Yes		Yes	Yes	Yes	Yes	Yes
If Yes, which **FORCE-based metric(s)** would you select?	Absolute peak force (N), Relative peak force (N.kg), Limb symmetry (L/R peak force) (%), Force at 100 ms (N), Force at 150 ms (N), Force at 200 ms (N), Force at 250 ms (N)	Net peak force (N), Relative peak force (N.kg), Force at 100 ms (N), Force at 250 ms (N)	Absolute peak force (N), Relative peak force (N.kg)	Absolute peak force (N), Net peak force (N), Relative peak force (N.kg), Force at 50 ms (N), Force at 100 ms (N), Force at 150 ms (N), Force at 200 ms (N), Force at 250 ms (N), Force at 300 ms (N), Non-injured limb - force at 50 ms (N), Mean net force from start to peak force		Net peak force (N), Relative peak force (N.kg), Force at 50 ms (N), Force at 100 ms (N), Force at 150 ms (N)	Net peak force (N), Relative peak force (N.kg), Limb symmetry (L/R peak force) (%), Force at 100 ms (N), Force at 200 ms (N)	Absolute peak force (N), Relative peak force (N.kg), Limb symmetry (L/R peak force) (%), Force at 100 ms (N), Force at 200 ms (N)	Net peak force (N), Relative peak force (N.kg), Force at 100 ms (N), Force at 150 ms (N)	Absolute peak force (N), Relative peak force (N.kg), Force at 100 ms (N), Force at 200 ms (N), Force at 300 ms (N)

Would you measure **RATE OF FORCE DEVELOPMENT metrics** from multi-joint isometric tests when informing **pre-course selection criteria**?	Yes	Yes	No	No		No	Yes	Yes	No	No
If Yes, which **RATE OF FORCE DEVELOPMENT (RFD) metric(s)** would you select?	Rate of force development 0-100 ms (N.s), Rate of force development 0-200 ms (N.s), Rate of force development 0-250 ms (N.s), PRFD at 20 ms moving window	Rate of force developmen t 0-100 ms (N.s), Rate of force developmen t 0-250 ms (N.s)					Rate of force development 0-50 ms (N.s), Rate of force development 0-100 ms (N.s), Rate of force development 0-200 ms (N.s), Rate of force development 0-300 ms (N.s)	Rate of force development 0-100 ms (N.s), Rate of force development 0-200 ms (N.s)		
Would you measure **IMPULSE metrics** from multi-joint isometric tests when informing **pre-course selection criteria**?	Yes	Yes	No	Yes		No	Yes	Yes	No	No
If yes, which **IMPULSE metric(s)** would you select?	Impulse 0-100 ms (N.s), Impulse 0-200 ms (N.s), Impulse 0-250 ms (N.s)	Impulse 0-100 ms (N.s), Impulse 0-250 ms (N.s), Net impulse 0-100 ms (N.s), Net impulse 0-250 ms (N.s)		Net impulse 0-50 ms (N.s), Net impulse 0-100 ms (N.s), Net impulse 0-150 ms (N.s), Net impulse 0-200 ms (N.s), Net impulse 0-250 ms (N.s)			Net impulse 0-50 ms (N.s), Net impulse 0-100 ms (N.s), Net impulse 0-200 ms (N.s), Net impulse 0-300 ms (N.s)	Impulse 0-100 ms (N.s), Impulse 0-200 ms (N.s)		
Would you measure **TIME-BASED metrics** from multi-joint isometric tests when informing **pre-course selection criteria**?	Yes	Yes	No	Yes		No	Yes	Yes	No	No

If yes, which **TIME-BASED metric(s)** would you select?	Time to peak force (s), Time to PRFD	Time to peak force (s)		Time to peak force (s)			Time to peak force (s)	Time to peak force (s)			
From the list of **multi-joint isometric test** metrics identified above, which **FIVE** would you consider the **most essential** to measure to inform **pre-course selection criteria**?<br><br>**Please list your FIVE answers below** (answers do not have to be in order of preference):	Relative peak force (N.kg), Impulse 0-200 ms (N.s), Limb symmetry (L/R peak force) (%), Force at 200 ms (N),Force at 100 ms (N)	net peak force; relative peak force; Time to peak force; RFD 100; RFD 250	Only 2 identified above	Peak net force, time to peak force, mean net force from start to peak force, force at 100 ms, force at 200 ms		Net force, net force/BM, net force at 50, 100, 150ms	1. Relative peak force; 2. Limb symmetry - peak force (%); 3. RFD 0-100ms; 4. Impulse 0-200ms; 5. Time to peak force	Relative peak force, limb asymmetry LR peak force, RFD 200ms, impulse 200 ms, time to peak force	Iso Squat or MTP Net Force, Relative Net Force, Force at 100ms or 150ms	Absolute and relative peak force, force @ 100 and 300 ms

Table S3. Single Joint Isometric tests

Questions	Responder									
	**1**	**2**	**3**	**4**	**5**	**6**	**7**	**8**	**9**	**10**
Do you consider force plate derived **Single Joint isometric** muscle testing appropriate / useful to inform **pre-course selection criteria**?	No	No	Yes	No		No	Yes	Yes	No	No
Which **single joint isometric tests** do you consider appropriate / useful to inform **pre-course selection criteria**?			Isometric 90/20 hamstring test, Isometric 'standing' plantar flexion test (unilateral)				Isometric 90/90 hamstring test, Isometric 30/30 hamstring test, Isometric 'seated' plantar flexion test (unilateral)	Isometric 90/90 hamstring test, Isometric 'seated' plantar flexion test (unilateral)		
Would you measure **FORCE related metrics** from single-joint isometric tests when informing **pre-course selection criteria**?			Yes				Yes	Yes		

If yes, which **FORCE metric(s)** would you select?			Absolute peak force (N), Relative peak force (N.kg)				Absolute peak force (N), Relative peak force (N.kg), Limb symmetry (L/R peak force) (%), Force at 100 ms (N), Force at 200 ms (N), Force at 300 ms (N), Injured limb - force at 100 ms (N), Injured limb - force at 200 ms (N), Injured limb - force at 300 ms (N), Non-injured limb - force at 100 ms (N), Non-injured limb - force at 200 ms	Absolute peak force (N), Relative peak force (N.kg), Limb symmetry (L/R peak force) (%), Force at 100 ms (N), Force at 200 ms (N)		
Would you measure **RATE OF FORCE DEVELOPMENT metrics** from single-joint isometric tests when informing **pre-course selection criteria**?			No				Yes	Yes		
If yes, which **RATE OF FORCE DEVELOPMENT (RFD) metric(s)** would you select?							Rate of force development 0-100 ms (N.s), Rate of force development 0-200 ms (N.s), Rate of force development 0-300 ms (N.s)	Rate of force development 0-100 ms (N.s), Rate of force development 0-200 ms (N.s)		

Would you measure **IMPULSE metrics** from single-joint isometric tests when informing **pre-course selection criteria**?			No				Yes	Yes		
If yes, which **IMPULSE metric(s)** would you select?							Impulse 0-100 ms (N.s), Impulse 0-200 ms (N.s), Impulse 0-300 ms (N.s)	Impulse 0-100 ms (N.s), Impulse 0-200 ms (N.s)		
Would you measure **TIME-BASED metrics** from single-joint isometric tests when informing **pre-course selection criteria**?			No				Yes	Yes		
If yes, which **TIME-BASED metric(s)** would you select?							Time to peak force (s)	Time to peak force (s)		
From the list of metrics identified above, which **FIVE** would you consider the **most essential** to measure to inform **pre-course selection criteria**?<br><br>**Please list your FIVE answers below** (answers do not have to be in order of preference):			Abs and Rel PF				1. Relative peak force; 2. Limb symmetry - peak force (%); 3. RFD 0-100ms; 4. Impulse 0-300ms; 5. Time to peak force	absolute peak force, relative peak force, limb symmetry, RFD 200ms, impulse 200ms		

Table S4. Countermovement jump

Questions	Responder 1	2	3	4	5	6	7	8	9	10
Do you consider the **countermovement jump** an appropriate / useful test to inform **pre-course selection criteria**?	Yes	Yes	Yes	No	Yes	Yes	Yes	Yes	Yes	Yes
Would you measure **OUTCOME metrics** from the countermovement jump test when informing **pre-course selection criteria**?	Yes	Yes	Yes		Yes	Yes	Yes	Yes	Yes	Yes
If yes, which **OUTCOME metric(s)** would you select?	Jump height (cm), Take off velocity (m.s)	Jump height (cm), Jump momentum (kg*m.s), Take off velocity (m.s), Reactive strength index (modified)	Jump height (cm), Reactive strength index (modified)		Jump height (cm), Reactive strength index (modified)	Jump height (cm), Jump momentum (kg*m.s)	Jump height (cm), Take off velocity (m.s), Reactive strength index (modified)	Jump height (cm), Take off velocity (m.s), Reactive strength index (modified)	Jump height (cm), Take off velocity (m.s), Reactive strength index (modified)	Jump height (cm), Take off velocity (m.s), Flight time (s)
Would you measure **FORCE metrics** from the countermovement jump test when informing **pre-course selection criteria**?	Yes	Yes	Yes		Yes	Yes	Yes	Yes	Yes	Yes

If yes, which **FORCE metric(s)** would you select?	Average relative braking/eccentric force (N.kg), Peak relative braking/eccentric force (N.kg), L/R average braking/eccentric force (%), L/R peak braking/eccentric force (%), Peak relative landing force (N.kg), Average relative propulsive/concentric force (N.kg), Peak relative propulsive/concentric force (N.kg)	L/R average propulsive/concentric force (%), L/R peak propulsive/concentric force (%), Average propulsion/concentric force (N), Peak net propulsive/concentric force (N), Average relative propulsive/concentric force (N.kg), Peak relative propulsive/concentric force (N.kg)	Average braking/eccentric force (N), Average propulsion/concentric force (N)		Peak relative landing force (N.kg)	Average braking/eccentric force (N), Average relative propulsive/concentric force (N.kg)	Average braking/eccentric force (N), Peak relative braking/eccentric force (N.kg), Injured limb - peak braking/eccentric force (N), Non-injured limb - peak braking/eccentric force (N), Peak relative landing force (N.kg), Injured limb - average landing force (N), Non-injured limb - average landing force (N), L/R peak landing force (%), Injured limb - force at peak propulsive/concentric force (N), Non-injured limb - force at peak propulsive/concentric force (N), L/R peak	Peak landing force (N), Peak braking/eccentric force (N), Average relative braking/eccentric force (N.kg), L/R peak braking/eccentric force (%), L/R peak landing force (%), Average propulsion/concentric force (N), Peak propulsive/concentric force (N)	Average braking/eccentric force (N), Peak braking/eccentric force (N), Force at min displacement (N)	Peak braking/eccentric force (N), Peak propulsive/concentric force (N)
Would you measure **POWER metrics** from the countermovement jump test when informing **pre-course selection criteria**?	Yes	No	No		Yes	No	Yes	Yes	Yes	No

If yes, which **POWER metric(s)** would you select?	Peak relative propulsive/concentric power (W.kg), Average propulsive/concentric power (W), Peak relative braking/eccentric power (W.kg), Average relative braking/eccentric power (W.kg)				Average relative breaking/eccentric power (W.kg)		Peak propulsive/concentric power (W), Peak relative propulsive/concentric power (W.kg), Peak braking/eccentric power (W), Peak relative braking/eccentric power (W.kg)	Peak propulsive/concentric power (W), Average propulsive/concentric power (W), Peak braking/eccentric power (W), Average braking/eccentric power (W)	Net peak propulsive/concentric power (W)	
Would you measure **VELOCITY metrics** from the countermovement jump test when informing **pre-course selection criteria**?	Yes	Yes	No		No	Yes	Yes	Yes	No	Yes
If yes, which **VELOCITY metric(s)** would you select?	Average propulsive/concentric velocity (m.s), Peak braking/eccentric velocity (m.s)	Average propulsive/concentric velocity (m.s)				Peak braking/eccentric velocity (m.s)	Average propulsive/concentric velocity (m.s), Peak braking/eccentric velocity (m.s)	Average propulsive/concentric velocity (m.s), Average braking/eccentric velocity (m.s)		Peak braking/eccentric velocity (m.s)
Would you measure **RATE OF FORCE DEVELOPMENT metrics** from the countermovement jump test when informing **pre-course selection criteria**?	Yes	Yes	No		No	Yes	Yes	Yes	Yes	No
If yes, which **RATE OF FORCE DEVELOPMENT (RFD) metric(s)** would you select?	L/R average breaking/eccentric RFD (%), Braking/eccentric RFD (N.s)	Braking/eccentric RFD (N.s)				Braking/eccentric RFD (N.s)	Injured limb - average braking/eccentric RFD, L/R average breaking/eccentric RFD (%), Braking/eccentric RFD (N.s)	L/R average breaking/eccentric RFD (%), Braking/eccentric RFD (N.s)	Braking/eccentric RFD (N.s)	

Would you measure **IMPULSE metrics** from the countermovement jump test when informing **pre-course selection criteria**?	Yes	Yes	No		Yes	No	Yes	Yes	No	No
If yes, which **IMPULSE metric(s)** would you select?	Impulse ratio, L/R braking/eccentric impuse index (%), Relative braking/eccentric impulse (N.s), L/R landing impulse index (%), Relative propulsive/concentric impulse (N.s.kg)	Impulse ratio, L/R braking/eccentric impuse index (%), Braking/eccentric net impulse (N.s), Positive net impulse (N.s), Propulsive/concentric net impulse (N.s), Relative propulsive/concentric net impulse (N.s.kg)			Impulse ratio		Relative braking/eccentric impulse (N.s), Braking/eccentric impulse (N.s), Braking/eccentric net impulse (N.s), L/R landing impulse index (%), L/R propulsive/concentric impulse index (%), Propulsive/concentric impulse (N.s), Relative propulsive/concentric impulse (N.s.kg)	Braking/eccentric impulse (N.s), Propulsive/concentric impulse (N.s)		
Would you measure **TIME-BASED metrics** from the countermovement jump test when informing **pre-course selection criteria**?	Yes	Yes	Yes		Yes	Yes	Yes	Yes	Yes	Yes

If yes, which **TIME-BASED metric(s)** would you select?	Time to takeoff / contraction time / movement time(s), Braking/eccentric phase duration (s), Propulsive/concentric phase duration (s), Time to stabilisation (s), Time to peak power (s)	Time to takeoff / contraction time / movement time(s), Braking/eccentric phase duration (s), Propulsive/concentric phase duration (s), Time to peak force (s), Propulsive/concentric force duration (s)	Braking/eccentric phase duration (s), Propulsive/concentric phase duration (s)		Time to takeoff / contraction time / movement time(s)	Time to takeoff / contraction time / movement time(s), Braking/eccentric phase duration (s), Propulsive/concentric force duration (s)	Time to takeoff / contraction time / movement time(s), Braking/eccentric phase duration (s), Time to stabilisation (s), Time to peak force (s), Propulsive/concentric force duration (s)	Time to takeoff / contraction time / movement time(s), Unweighted phase duration (s), Braking/eccentric phase duration (s), Propulsive/concentric phase duration (s), Time to peak force (s)	Time to takeoff / contraction time / movement time(s), Unweighted phase duration (s), Braking/eccentric phase duration (s), Propulsive/concentric phase duration (s)	Time to takeoff / contraction time / movement time(s)
Would you measure **OTHER metrics** from the countermovement jump test when informing **pre-course selection criteria**?	Yes	No	Yes		Yes	Yes	Yes	Yes	Yes	Yes

If yes, which **OTHER metric(s)** derived would you select?	Countermovement depth (m), Landing performance index		Countermovement depth (m)		Force-velocity AUC (N.m.s.kg)), Landing performance index	Countermovement depth (m)	Countermovement depth (m), Force-velocity AUC (N.m.s.kg))	Countermovement depth (m)	Countermovement depth (m)	Countermovement depth (m), Development of infantry specific spatial parametric mapping of the force time curve related to injury risk
From the list of metrics identified above, which **FIVE** would you consider the **most essential** to measure to inform **pre-course selection criteria**?<br><br>**Please list your FIVE answers below** (answers do not have to be in order of preference):	Jump Height (cm), Peak relative propulsive/concentric power (W.kg), L/R average propulsive/concentric impulse index (%), Braking/eccentric RFD (N/s) , Time to stabilisation (s)	JH, Jump momentum; Time to takeoff; peak force;	Jump height<br>RSIm<br>Ave Braking F<br>Ave Prop F<br>CoM displacement		Jump height (cm)<br>Reactive strength index (modified)<br>Peak relative landing force (N.kg)<br>Time to takeoff / contraction time / movement time(s)<br>Impulse ratio	jump height, jump momentum, contraction time, eccentric peak velocity, average braking force.	1. RSI modified; 2. L/R peak braking force; 3. Braking/eccentric RFD; 4. Relative propulsive net impulse; 5. Peak relative propulsive power	jump height, reactive strength index modified, peak concentric force, peak eccentric force, countermovement depth	jump height, Braking RFD, Peak Braking Force RSI-mid, Net Propulsive Power	Countermovement depth, peak propulsive and eccentric force, Flight time:Contraction time

Table S5. Squat jump

Questions	Responder									
	1	2	3	4	5	6	7	8	9	10
Do you consider the **squat jump** an appropriate / useful test to inform **pre-course selection criteria**?	Yes	Yes	No	No	No	No	Yes	Yes	No	No
Would you measure **OUTCOME metrics** from the squat jump test when informing **pre-course selection criteria**?	Yes	Yes					Yes	Yes		
If yes, which **OUTCOME metric(s)** would you select?	Jump height (cm), Take off velocity (m.s)	Jump height (cm), Jump momentum (kg*m.s), Take off velocity (m.s), Time to takeoff / contraction time / movement time (s)					Jump height (cm), Take off velocity (m.s), Time to takeoff / contraction time / movement time (s)	Jump height (cm), Take off velocity (m.s)		
Would you measure **FORCE metrics** from the squat jump test when informing **pre-course selection criteria**?	Yes	Yes					Yes	Yes		

If yes, which **FORCE metric(s)** would you select?	L/R average landing force (%), L/R peak landing force (%), Average landing force (N.m), Peak landing force (N), Peak relative landing force (N.kg), L/R average propulsive/concentric force (%), L/R peak propulsive/concentric force (%), Average propulsion/concentric force (N), Average relative propulsive/concentric force (N.kg), Peak propulsive/concentric force (N), Peak relative propulsive/concentric force (N.kg)	L/R average propulsive/concentric force (%), L/R peak propulsive/concentric force (%), Peak net propulsive/concentric force (N), Peak relative propulsive/concentric force (N.kg)					Injured limb - force at peak propulsive/concentric force (N), Non-injured limb - force at peak propulsive/concentric force (N), L/R peak propulsive/concentric force (%), Average relative propulsive/concentric force (N.kg), Peak relative propulsive/concentric force (N.kg)	L/R peak landing force (%), Peak landing force (N), Average propulsion/concentric force (N), Peak propulsive/concentric force (N)		
Would you measure **POWER metrics** from the squat jump test when informing **pre-course selection criteria**?	Yes	No					Yes	Yes		

If yes, which **POWER metric(s)** would you select?	Peak propulsive/concentric power (W), Peak relative propulsive/concentric power (W.kg), Average propulsive/concentric power (W), Average relative propulsive/concentric power (W.kg)						Peak propulsive/concentric power (W), Peak relative propulsive/concentric power (W.kg), Average propulsive/concentric power (W), Average relative propulsive/concentric power (W.kg)	Peak propulsive/concentric power (W), Average propulsive/concentric power (W)		
Would you measure **VELOCITY metrics** from the squat jump test when informing **pre-course selection criteria**?	Yes	Yes					Yes	Yes		
If yes, which **VELOCITY metric(s)** derived would you select?	Average propulsive/concentric velocty (m.s)	Average propulsive/concentric velocty (m.s)					Average propulsive/concentric velocty (m.s)	Average propulsive/concentric velocty (m.s)		
Would you measure **IMPULSE metrics** from the squat jump test when informing **pre-course selection criteria**?	Yes	Yes					Yes	Yes		

If yes, which **IMPULSE metric(s)** derived would you select?	L/R landing impulse index (%), Relative propulsive/concentric impulse (N.s.kg), Relative propulsive/concentric net impulse (N.s.kg), Propulsive/concentric net impulse (N.s), Landing impulse (N.s)	L/R propulsive/concentric impulse index (%), Relative propulsive/concentric net impulse (N.s.kg)					Relative propulsive/concentric net impulse (N.s.kg), Propulsive/concentric impulse (N.s)	Propulsive/concentric impulse (N.s)		
Would you measure **TIME-BASED metrics** from the squat jump test when informing **pre-course selection criteria**?	Yes	Yes					Yes	Yes		
If yes, which **TIME-BASED metric(s)** would you select?	Time to stabilisation (s), Time to peak force (s), Time to peak power (s), Propulsive phase duration (s)	Time to peak force (s), Propulsive phase duration (s)					Time to peak force (s), Time to peak power (s), Propulsive phase duration (s)	Time to peak force (s), Propulsive phase duration (s)		
Would you measure **OTHER metrics** from the squat jump test when informing **pre-course selection criteria**?	Yes	No					No	No		
If yes, which **OTHER metric(s)** derived would you select?	Landing performance index									

From the list of metrics identified above, which **FIVE** would you consider the **most essential** metrics to measure to inform **pre-course selection criteria**?<br><br>**Please list your FIVE answers below** (answers do not have to be in order of preference):	Jump Height, Peak Relative Propulsive/Concentric Power (W/kg), Time to Stabilisation (s),L/R Propulsive/Concentric Impulse Index (%), Peak Relative Landing Force (N/kg)	JH; jump momentum; LR propulsive force%; LR propulsive impulse%; Time to takeoff					1. Jump height; 2. Peak relative propulsive force; 3. Relative propulsive impulse; 4. Relative propulsive power; 5. Time to peak force	rsi modified, jump height, concentric phase duration, peak concetric force, peak concetric power		

Table S6. Drop jump

Questions	Responder									
	**1**	**2**	**3**	**4**	**5**	**6**	**7**	**8**	**9**	**10**
Do you consider the **drop jump test** an appropriate / useful test to inform **pre-course selection criteria**?	Yes	Yes	Yes	Yes	Yes	Yes	Yes	No	Yes	Yes
Would you measure **OUTCOME metrics** from the drop jump test when informing **pre-course selection criteria**?	Yes	Yes	Yes	Yes	Yes	Yes	Yes		Yes	Yes
If yes, which **OUTCOME metric(s)** would you select?	Reactive strength index, Jump height (cm), Flight time (s), Takeoff velocity (m.s)	Reactive strength index	Reactive strength index, Jump height (cm), Flight time (s), Drop height (cm)	Reactive strength index, Jump momentum (kg*m.s), Jump height (cm), Drop height (cm)	Reactive strength index, Jump height (cm), Drop height (cm)	Reactive strength index, Jump height (cm)	Reactive strength index, Jump height (cm), Takeoff velocity (m.s)		Reactive strength index, Jump height (cm), Drop height (cm)	Reactive strength index, Jump height (cm), Takeoff velocity (m.s)
Would you measure **FORCE metrics** from the drop jump test when informing **pre-course selection criteria**?	Yes	Yes	Yes	Yes	No	No	Yes		Yes	Yes

If yes, which **FORCE metric(s)** would you select?	L/R average braking/eccentric force (%), L/R peak braking/eccentric force (%), Average relative braking/eccentric force (N.kg), Peak relative braking/eccentric force (N.kg), Average landing force (N), Peak landing force (N), L/R average propulsive/concentric force (%), L/R peak propulsive/concentric force (%), Average propulsive/concentric force (N), Peak propulsive/concentric force (N)	L/R average braking/eccentric force (%), L/R peak braking/eccentric force (%), Average relative braking/eccentric force (N.kg), Peak relative braking/eccentric force (N.kg), L/R average propulsive/concentric force (%), L/R peak propulsive/concentric force (%)	Average braking/eccentric force (N), Average propulsive/concentric force (N)	Average braking/eccentric force (N), Peak braking/eccentric force (N), Average relative braking/eccentric force (N.kg), Peak relative braking/eccentric force (N.kg), Average landing force (N), Peak landing force (N), L/R average propulsive/concentric force (%), Average propulsive/concentric force (N), Peak propulsive/concentric force (N), Force at min displacement (N), Average braking net force, average propulsive net force			L/R peak braking/eccentric force (%), Average braking/eccentric force (N), Peak relative braking/eccentric force (N.kg), L/R peak landing force (%), Relative peak landing force (N), L/R peak propulsive/concentric force (%), Peak propulsive/concentric force (N)		Peak braking/eccentric force (N), Force at min displacement (N)	Peak propulsive/concentric force (N)
Would you measure **POWER metrics** from the drop jump test when informing **pre-course selection criteria**?	Yes	No	No	No	No	No	Yes		No	No

If yes, which **POWER metric(s)** would you select?	Average relative braking/eccentric power (W.kg), Peak relative braking/eccentric power (W.kg), Average relative propulsive/concentric power (W.kg), Peak relative propulsive/concentric power (W.kg						Average relative braking/eccentric power (W.kg), Peak relative braking/eccentric power (W.kg), Peak relative propulsive/concentric power (W.kg, Average propulsive/concentric power (W), Peak			
Would you measure **VELOCITY metrics** from the drop jump test when informing **pre-course selection criteria**?	Yes	Yes	No	Yes	No	No	Yes		No	Yes
If yes, which **VELOCITY metric(s)** would you select?	Average propulsive/concentric velocity (m.s), Average braking/eccentric velocity (m.s)	Average propulsive/concentric velocity (m.s), Average braking/eccentric velocity (m.s)		Average propulsive/concentric velocity (m.s), Average braking/eccentric velocity (m.s)			Average propulsive/concentric velocity (m.s), Average braking/eccentric velocity (m.s)			peak braking/eccentric velocity
Would you measure **RATE OF FORCE DEVELOPMENT (RFD) metrics** from the drop jump test when informing **pre-course selection criteria**?	Yes	Yes	No	No	No	No	Yes		No	No
If yes, which **RATE OF FORCE DEVELOPMENT metric(s)** would you select?	L/R average braking/eccentric RFD (%), Braking/eccentric RFD (N.s)	L/R average braking/eccentric RFD (%)					L/R average braking/eccentric RFD (%), Braking/eccentric RFD (N.s)			

Would you measure **IMPULSE metrics** from the drop jump test when informing **pre-course selection criteria**?	Yes	Yes	No	Yes	Yes	No	Yes		No	No
If yes, which **IMPULSE metric(s)** would you select?	L/R braking/eccentric impulse index (%), Relative braking/eccentric impulse (N.s.kg), L/R landing impulse index (%), relative propulsive/concentric impulse (N.s.kg), Impulse ratio	L/R braking/eccentric impulse index (%), Relative braking/eccentric net impulse (N.s.kg), Relative propulsive/concentric net impulse (N.s.kg), Impulse ratio		Braking/eccentric net impulse (N.s), Propulsive/concentric net impulse (N.s)	Positive net impulse (N.s)		L/R braking/eccentric impulse index (%), Relative braking/eccentric impulse (N.s.kg), relative propulsive/concentric impulse (N.s.kg), Propulsive/concentric impulse (N.s), Impulse ratio			
Would you measure **TIME-BASED metrics** from the drop jump test when informing **pre-course selection criteria**?	Yes	Yes	Yes	Yes	Yes	Yes	Yes		Yes	Yes
If yes, which **TIME-BASED metric(s)** would you select?	Time to peak braking/eccentric force (s), Braking/eccentric phase duration (s), Propulsive/concentric phase duration (s), Time to stabilisation (s)	Braking/eccentric phase duration (s), Propulsive/concentric phase duration (s)	Braking/eccentric phase duration (s), Propulsive/concentric phase duration (s)	Time to peak braking/eccentric force (s), Braking/eccentric phase duration (s), Propulsive/concentric phase duration (s), Landing phase duration (s)	Contact Time	Braking/eccentric phase %, Landing phase duration (s)	Time to peak braking/eccentric force (s), Braking/eccentric phase duration (s), Propulsive/concentric phase duration (s), Time to stabilisation (s)		Braking/eccentric phase duration (s), Propulsive/concentric phase duration (s)	Time to peak braking/eccentric force (s), Propulsive/concentric phase duration (s), Time to stabilisation (s)

Would you measure **OTHER metrics** from the drop jump test when informing **pre-course selection criteria**?	Yes	No	Yes	Yes	Yes	Yes	Yes		Yes	Yes
If yes, which **OTHER metric(s)** would you select?	Spring like correlation, Landing stiffness (N.m), Stiffness during rebound phase (N.m), Countermovement depth (cm)	Landing stiffness (N.m)	Spring like correlation, Countermovement depth (cm)	Countermovement depth (cm)	Spring like correlation	Spring like correlation	Spring like correlation, Landing stiffness (N.m), Landing performance index		Landing stiffness (N.m)	Landing stiffness (N.m), Countermovement depth (cm)
From the list of metrics identified above, which **FIVE** would you consider the **most essential** metrics to measure to inform the **pre-course selection criteria**?<br><br>**Please list your FIVE answers below** (answers do not have to be in order of preference):	Reactive Strength Index (RSI), Peak Propulsive/Concentric Power (W/kg - relative), L/R Braking/Eccentric Impulse Index (%), Time to Stabilisation (s), Landing Stiffness (N/m)	RSI; LR braking force; LR propulsive force%; Braking and propulsive phase duration	RSI<br>Jump height<br>Flight time<br>Ave braking F<br>Ave Prop F	Drop height, jump height, average braking net force, braking time, propulsive time	Reactive strength index<br>Jump height (cm)<br>Contact Time<br>Spring like correlation<br>Positive net impulse (N.s)	RSI, JH (flight time), contact time, spring like correlation.	1. RSI; 2. Landing stiffness; 3. Average propulsive/concentric power; 4. Spring-like correlation; 5. Braking/eccentric RFD		RSI, Jump Height, braking+ propulsive phase duration, Peak Braking force	Countermovement depth, peak propulsive force, landing stiffness
What box height do you feel is appropriate to use when conducting a **drop vertical jump** test to inform **pre-course selection criteria?**	Bilateral DVJ - 20cm, Bilateral DVJ - 30cm, Bilateral DVJ - 40cm, Unilateral DVJ - 20cm, Unilateral DVJ - 30cm	Bilateral DVJ - 40cm, Unilateral DVJ - 20cm	Bilateral DVJ - 30cm	Bilateral DVJ - 20cm, Bilateral DVJ - 30cm, Bilateral DVJ - 40cm, Bilateral DVJ - 50cm, Bilateral DVJ - 60cm	Bilateral DVJ - 30cm	Bilateral DVJ - 30cm	Bilateral DVJ - 30cm, Unilateral DVJ - 20cm		Bilateral DVJ - 30cm, Bilateral DVJ - 40cm	Bilateral DVJ - 40cm, Bilateral DVJ - 50cm

Table S7. CMJ Rebound Jump

Questions	Responder									
	1	2	3	4	5	6	7	8	9	10
Do you believe **CMJ rebound jump tests** are appropriate / useful to inform **pre-course selection criteria**?	Yes	Yes	No	Yes	Yes	No	Yes	No	No	No
Would you measure **OUTCOME metrics** from the CMJ Rebound Jump test when informing **pre-course selection criteria**?	Yes	Yes		Yes	Yes		Yes			
If yes, which **OUTCOME metric(s)** would you select?	CMJ - modified reactice strength index (mRSI), Rebound - Reactive strength index (RSI), CMJ - jump height (cm), Rebound - jump height (cm)	Rebound - Reactive strength index (RSI), Rebound - jump momentum (kg*m.s), Rebound - jump height (cm)		CMJ - modified reactice strength index (mRSI), Rebound - Reactive strength index (RSI), CMJ - jump momentum (kg*m.s), Rebound - jump momentum (kg*m.s), CMJ - jump height (cm), Rebound - jump height (cm)	CMJ - modified reactice strength index (mRSI), Rebound - Reactive strength index (RSI), CMJ - jump height (cm), Rebound - jump height (cm)		CMJ - modified reactice strength index (mRSI), Rebound - Reactive strength index (RSI), CMJ - jump height (cm), Rebound - jump height (cm)			
Would you measure **FORCE metrics** from the CMJ Rebound Jump test when informing **pre-course selection criteria**?	Yes	Yes		Yes	No		Yes			

If yes, which **FORCE metric(s)** would you select?	CMJ - average braking/eccentric force (N), CMJ - peak braking/eccentric force (N), CMJ - average relative braking/eccentric force (N.kg), CMJ - peak relative braking/eccentric force (N.kg), L/R average landing force (%), L/R peak landing force (%), CMJ - average propulsive/concentric force (N), CMJ - peak propulsive/concentric force (N), CMJ - average relative propulsive/concentric force (N.kg), CMJ - peak relative propulsive/concentric force (N.kg), Rebound - average propulsive/conc	Rebound - L/R average braking/eccentric force (%), Rebound - average relative braking/eccentric force (N.kg), Rebound - peak relative braking/eccentric force (N.kg), Rebound - average relative propulsive/concentric force (N.kg), Rebound - peak relative propulsive/concentric force (N.kg)		CMJ - average braking/eccentric force (N), CMJ - peak braking/eccentric force (N), Rebound - average braking/eccentric force (N), Rebound - peak braking/eccentric force (N), Rebound - time to peak braking/eccentric force (s), Average landing force (N), Peak landing force (N), CMJ - average propulsive/concentric force (N), CMJ - peak propulsive/concentric force (N), Rebound - average propulsive/concentric force (N), Rebound - peak propulsive/concentric force (N), CMJ - force at			Rebound - L/R average braking/eccentric force (%), Rebound - peak relative braking/eccentric force (N.kg), Rebound - time to peak braking/eccentric force (s), Rebound - L/R average propulsive/concentric force (%), Rebound - peak relative propulsive/concentric force (N.kg)			
Would you measure **POWER metrics** from the CMJ Rebound Jump test when informing **pre-course selection criteria**?	Yes	No		No	No		Yes			

If yes, which **POWER metric(s)** would you select?	CMJ - average relative braking/eccentric power (W.kg), CMJ - peak relative braking/eccentric power (W.kg), Rebound - average relative braking/eccentric power (W.kg), Rebound - peak relative braking/eccentric power (W.kg), CMJ - average relative propulsive/concentric power (W.kg), CMJ - peak relative propulsive/concentric power (W.kg), Rebound - average relative propulsive/concentric power (W.kg), Rebound - peak relative propulsive/concentric power (W.kg)						CMJ - peak relative braking/eccentric power (W.kg), Rebound - peak relative braking/eccentric power (W.kg), CMJ - peak relative propulsive/concentric power (W.kg), Rebound - peak relative propulsive/concentric power (W.kg)			

Would you measure **RATE OF FORCE DEVELOPMENT (RFD) metrics** from the CMJ Rebound Jump test when informing **pre-course selection criteria**?	Yes	Yes		No	No		Yes			
If yes, which **RATE OF FORCE DEVELOPMENT (RFD) metric(s)** would you select?	CMJ - braking/eccentric RFD (N.s)	CMJ - braking/eccentric RFD (N.s)					CMJ - braking/eccentric RFD (N.s)			
Would you measure **IMPULSE metrics** from the CMJ Rebound Jump test when informing **pre-course selection criteria**?	Yes	Yes		Yes	No		Yes			

If yes, which **IMPULSE metric(s)** would you select?	CMJ - relative braking/eccentric impulse (N.s.kg), CMJ - relative braking/eccentric net impulse (N.s.kg), Rebound - relative braking/eccentric impulse (N.s.kg), Rebound - relative braking/eccentric net impulse (N.s.kg), L/R Landing impulse index (%), CMJ - relative propulsive/concentric impulse (N.s.kg), CMJ - relative propulsive/concentric net impulse (N.s.kg), Rebound - relative propulsive/concentric impulse (N.s.kg), Rebound	Rebound - L/R braking/eccentric impulse index (%), Rebound - relative braking/eccentric net impulse (N.s.kg), Rebound - positive net impulse (N.s), Rebound - relative propulsive/concentric net impulse (N.s.kg), CMJ - impulse ratio, Rebound - impulse ratio		Rebound - braking/eccentric net impulse (N.s), CMJ - propulsive/concentric net impulse (N.s), Rebound - propulsive/concentric net impulse (N.s)			CMJ - relative braking/eccentric impulse (N.s.kg), Rebound - relative braking/eccentric impulse (N.s.kg), CMJ - relative propulsive/concentric impulse (N.s.kg), Rebound - relative propulsive/concentric impulse (N.s.kg), Rebound - impulse ratio			
Would you measure **TIME-BASED metrics** from the CMJ Rebound Jump test when informing **pre-course selection criteria**?	Yes	Yes		Yes	Yes		Yes			

If yes, which **TIME-BASED metric(s)** would you select?	CMJ - time to takeoff (s), Rebound - ground contact time (s), Time to stabilisation (s), Landing phase duration (s)	CMJ - time to takeoff (s), Rebound - time to takeoff (s), Rebound - ground contact time (s)		CMJ - time to takeoff (s), Rebound - time to takeoff (s), Rebound - ground contact time (s), Landing phase duration (s)	CMJ - time to takeoff (s), Rebound - ground contact time (s)		CMJ - time to takeoff (s), Rebound - ground contact time (s), Time to stabilisation (s)			
Would you measure **OTHER metrics** from the CMJ Rebound Jump test when informing **pre-course selection criteria**?	Yes	No		Yes	Yes		Yes			
If yes, which **OTHER metric(s)** would you select?	Landing stiffness (N.m), Countermovement depth (cm), Rebound - countermovement depth (cm), Landing performance index	Landing stiffness (N.m)		Countermovement depth (cm), Rebound - countermovement depth (cm)	Rebound - spring like correlation		Rebound - spring like correlation, Rebound - stiffness (N.m)			

From the list of metrics identified above, which **<u>TEN</u>** would you consider the **most essential** metrics to measure to inform the **pre-course selection criteria**?<br><br>**Please list your <u>TEN</u> answers below** (answers do not have to be in order of preference):	CMJ - jump height (cm), Rebound - Reactive Strength Index (RSI), CMJ - peak relative propulsive/concentric power (W.kg), Rebound - ground contact time (s), CMJ - peak relative braking/eccentric force (N.kg), Rebound - peak relative braking/eccentric force (N.kg), CMJ - L/R average propulsive/concentric force (%), Rebound - L/R average braking/eccentric force (%), Time to stabilisation (s):, Rebound - stiffness (N.m)	Reactive strength index; rebound JH; rebound braking impulse; rebound propulsive impulse; time to takeoff		Countermovement depth, rebound depth, CMJ time to takeoff, CMJ braking time, rebound time to takeoff, rebound braking time, CMJ braking impulse, CMJ propulsive impulse, rebound braking impulse, rebound propulsive impulse	CMJ - modified reactice strength index (mRSI)<br>Rebound - Reactive strength index (RSI)<br>CMJ - jump height (cm)<br>Rebound - jump height (cm)<br>CMJ - time to takeoff (s)<br>Rebound - ground contact time (s)<br>Rebound - spring like correlation		1. Rebound - RSI; 2. Rebound - ground contact time; 3. CMJ - RSI modified; 4. Rebound - braking/eccentric impulse; 5. Rebound - L/R propulsive/concentric force (%); 6. Rebound - spring-like correlation; 7. Rebound - relative braking/eccentric impulse; 8. Landing stiffness; 9. Rebound - jump height; 10. CMJ - jump height			

Table S8. Drop landing

Questions	Responder 1	2	3	4	5	6	7	8	9	10
Do you consider the **drop landing tasks** to be an appropriate / useful test to inform **pre-course selection criteria**?	Yes	No	No	No	No	No	Yes	No	No	No
Would you measure **OUTCOME metrics** from a drop landing task when informing **pre-course selection criteria**?	No						Yes			
If yes, which **OUTCOME metric(s)** would you select?							Drop height (cm)			
Would you measure **FORCE metrics** from the Drop Landing task when informing **pre-course selection criteria**?	Yes						Yes			
If yes, which **FORCE metric(s)** would you select?	L/R peak force (%), L/R average impact force (%), Average impact force (N), Peak force (N), peak relative force (N.kg), L/R average stabilisation force (%), Average stabilisation force (N)						Injured limb - force at peak force (N), Non-injured limb - force at peak force (N), L/R peak force (%), Peak force (N)			
Would you measure **POWER metrics** from the Drop Landing task when informing **pre-course selection criteria**?	Yes						Yes			

If yes, which **POWER metric(s)** would you select?	Average relative impact power (W.kg), Peak relative impact power (W.kg), Average relative stabilisation power (W.kg), Peak relative stabilisation power (W.kg)						Peak relative impact power (W.kg), Peak impact power (W), Average relative stabilisation power (W.kg), Peak relative stabilisation power (W.kg)			
Would you measure **VELOCITY metrics** from the Drop Landing task when informing **pre-course selection criteria**?	Yes						Yes			
If yes, which **VELOCITY metric(s)** would you select?	Average impact velocity (m.s), Average stabilisation velocity (m.s)						Average impact velocity (m.s), Average stabilisation velocity (m.s)			
Would you measure **RATE OF FORCE DEVELOPMENT (RFD) metrics** from the Drop Landing task when informing **pre-course selection criteria**?	Yes						Yes			
If yes, which **RATE OF FORCE DEVELOPMENT (RFD) metric(s)** would you select?	Impact RFD (N.s)						Impact RFD (N.s)			
Would you measure **TIME-BASED metrics** from the Drop Landing task when informing **pre-course selection criteria**?	Yes						Yes			

If yes, which **TIME-BASED metric(s)** would you select?	Impact phase duration (s), Impact phase %, Stabilisation phase duration (s), Time to stabilisation (s), Landing phase duration (s)						Impact phase duration (s), Time to stabilisation (s)				
Would you measure **OTHER metrics** from the Drop Landing task when informing **pre-course selection criteria**?	Yes						Yes				
If yes, which **OTHER metric(s)** would you select?	Stiffness (N.m), Landing performance index, Landing impulse (N.s)						Stiffness (N.m), Landing performance index				
From the list of metrics identified above, which **FIVE** would you consider the **most essential** metrics to measure to inform the **pre-course selection criteria**?<br><br>**Please list your FIVE answers below** (answers do not have to be in order of preference):	Time to Stabilisation (s), Peak Relative Force (N/kg), L/R Peak Force (%) , Stiffness (N/m), Peak Relative Impact Power (W/kg)						1. Time to stabilization; 2. Landing performance index; 3. L/R peak force (%); 4. Stiffness; 5. Relative peak landing force				

What box height do you feel is appropriate to use when conducting a **drop landing task** to inform **pre-course selection criteria?**	Bilateral landing - 20cm, Bilateral landing - 30cm, Bilateral landing - 40cm, Bilateral landing - 50cm, Unilateral landing - 20cm, Unilateral landing - 30cm, Unilateral landing - 40cm						Bilateral landing - 40cm, Bilateral landing - 50cm, Unilateral landing - 30cm			

Table S9. Multi Rebound Jump

Questions	Responder									
	**1**	**2**	**3**	**4**	**5**	**6**	**7**	**8**	**9**	**10**
Do you consider **multi rebound jump tests** to be appropriate / useful test to inform **pre-course selection criteria**?	Yes	Yes	No	No	No	No	Yes	No	No	Yes
Which **multi rebound jump/hop tests** appropriate to use to inform **pre-course selection criteria**?	10/5 rebound, Pogo jumps / hops, Bosco jump	10/5 rebound, 5/3 rebound					10/5 rebound, Pogo jumps / hops			10/5 rebound
Would you measure **OUTCOME metrics** from a multi-rebound jump/hop test when informing **pre-course selection criteria**?	Yes	Yes					Yes			Yes

If yes, which **OUTCOME metric(s)** would you select?	Peak jump reactive strength index (RSI), Peak RSI, Top 3 jumps/hops average RSI, Top 5 jumps/hops average RSI, Top 3 jumps/hops average jump height (cm), Top 5 jumps/hops average jump height (cm)	Top 3 jumps/hops average RSI, Top 3 jumps/hops peak RSI, Top 5 jumps/hops average RSI, Top 5 jumps/hops peak RSI, Average jump height (cm), Top 3 jumps/hops average jump height (cm), Top 5 jumps/hops average jump height (cm)					Top 3 jumps/hops average RSI, Top 3 jumps/hops average jump height (cm)			Top 3 jumps/hops peak RSI
Would you measure **FORCE metrics** from a multi-rebound jump/hop test when informing **pre-course selection criteria**?	Yes	Yes					Yes			Yes
If yes, which **FORCE metric(s)** would you select?	Average force (N), Peak force (N), L/R average force (%), L/R peak force (%)	Average force (N), L/R average force (%)					Average force (N), L/R average force (%)			Average force (N)
Would you measure **TIME-BASED metrics** from a multi-rebound jump/hop test when informing **pre-course selection criteria**?	Yes	Yes					Yes			No

If yes, which **TIME-BASED metric(s)** would you select?	Total contact time (s), Total flight time (s), Top 3 jumps/hops average contact time (s), Average contact time (s)	Top 3 jumps/hops average contact time (s), Top 5 jumps/hops average contact time (s), Average contact time (s)					Average contact time (s)			
From the list of metrics identified above, which **five** would you consider the **most essential** metrics to record to inform the **pre-course selection criteria**?<br><br>**Please list your FIVE answers below** (answers do not have to be in order of preference):	Average Reactive Strength Index (RSI), Average Contact Time (s), Peak Jump Reactive Strength Index (RSI), Peak Force (N) , L/R Average Force (%) (Limb Symmetry Index for Force)	top 3/5 RSI, Top 3/5 average jump height; top 3/5 average ground contact time					1. Average contact time; 2. Average force; 3. L/R average force (%); 4. Top 3 jumps RSI (average); 5. Top 3 jumps jump height (average)			Top 5 jumps/hops average contact time, Top 5 jumps/hops peak RSI, Peak jump reactive strength index (RSI)

Are there any other force plate tests not listed that you would consider to be appropriate / useful force plate test to inform **pre-course selection criteria**?<br><br>If yes, please provide the **NAME of the test** and your **top FIVE recommended metrics** derived from this test.							N/A			Not a test per se but a higher order statistical/mathematical analysis of the force/time data series from CMJ testing rather than the use of discrete metrics

Table S10. Other: Loaded jumps, ratio metrics

Questions	Responder 1	2	3	4	5	6	7	8	9	10
If portable force plates were to be integrated into UK Defence practice, there will likely be time constraints associated with their administration (for example, strict management of large numbers during annual fitness testing). If faced with a scenario where the practitioner is only provided with enough time to use **THREE force plate tests to inform pre-course selection criteria, which THREE tests would you recommend following an KNEE injury?**	Isometric midthigh pull, Countermovement jump, 5 / 3 rebound	Isometric midthigh pull, Squat jump, CMJ rebound jump	Isometric belt squat, Countermovement jump, Drop jump	Isometric midthigh pull, Countermovement jump, Drop jump	Countermovement jump, Drop jump, CMJ rebound jump	Isometric back squat, Drop jump, CMJ rebound jump	Isometric midthigh pull, Countermovement jump, Drop jump	Isometric midthigh pull, Countermovement jump, Squat jump	Isometric back squat, Countermovement jump, Drop jump	Isometric midthigh pull, Countermovement jump, Drop jump
Would you consider **loaded** force plate testing appropriate / useful to inform **pre-course selection criteria**?	Yes	Yes	No	No	Yes	No	No	No	Yes	Yes
Would you consider any of the following tests appropriate / useful to perform under **loaded conditions** to inform **pre-course selection criteria**?	CMJ, Squat jump	CMJ, Squat jump			CMJ		CMJ, Squat jump		Squat jump	CMJ
Would any of the following **ratio-based metrics** be considered useful to collect/analyse/report during **pre-course selection**?	Elastic utilisation ratio	Dynamic strength index/deficit		Dynamic strength index/deficit		Elastic utilisation ratio	Both can be beneficial	Elastic utilisation ratio	Dynamic strength index/deficit	Both

If you said yes to the use of the Dynamic Strength Index/Deficit, what tests and metrics would you use in your calculations?	Isometric Mid-Thigh Pull (IMTP), Countermovement Jump (CMJ), Squat Jump (SJ)	Mid thigh pull and squat jump	While their conceptual utility is strong, I'm not yet convinced of their practical application	It wouldn't be the same for each stage... where possible begin with the CMJ during/at the end of the early stage, progressing to the rebound jump for the mid stage, and then perhaps the drop jump for the late stage (or stick with the rebound jump if needed)			CMJ, IMTP, SJ	For the Dynamic Strength Index/Deficit, I would use:<br><br>Tests: Isometric mid-thigh pull (IMTP) or isometric squat for maximal isometric force, paired with a countermovement jump (CMJ) or squat jump (SJ) for dynamic force output.<br><br>Metrics: Peak force from IMTP (N) and peak force from CMJ/SJ (N) to calculate DSI (Dynamic Peak Force ÷ Isometric Peak Force).	Net Peak Isometric Force and CMJ Height or Net Propulsive Force.<br><br>NA.	IMTP peak force and CMJ peak propulsive force - same method at any time
